# Dehydrogenation of anhydrous methanol at room temperature by *o*-aminophenol-based photocatalysts

**DOI:** 10.1038/ncomms12333

**Published:** 2016-07-26

**Authors:** Masanori Wakizaka, Takeshi Matsumoto, Ryota Tanaka, Ho-Chol Chang

**Affiliations:** 1 Department of Applied Chemistry, Faculty of Science and Engineering, Chuo University, 1-13-27 Kasuga, Bunkyo-ku, Tokyo 112-8551, Japan

## Abstract

Dehydrogenation of anhydrous methanol is of great importance, given its ubiquity as an intermediate for the production of a large number of industrial chemicals. Since dehydrogenation of methanol is an endothermic reaction, heterogeneous or homogeneous precious-metal-based catalysts and high temperatures are usually required for this reaction to proceed. Here we report the photochemical dehydrogenation of anhydrous methanol at room temperature catalysed by *o*-aminophenol (apH_2_), *o*-aminophenolate (apH^−^) and the non-precious metal complex *trans*-[Fe^II^(apH)_2_(MeOH)_2_]. Under excitation at 289±10 nm and in the absence of additional photosensitizers, these photocatalysts generate hydrogen and formaldehyde from anhydrous methanol with external quantum yields of 2.9±0.15%, 3.7±0.19% and 4.8±0.24%, respectively, which are the highest values reported so far to the best of our knowledge. Mechanistic investigations reveal that the photo-induced formation of hydrogen radicals triggers the reaction.

## Abstract (web summary)

Photochemical dehydrogenation of anhydrous methanol is a relatively low temperature hydrogen generation process. Here, the authors report a series of compounds for room temperature anhydrous methanol dehydrogenation, together with the mechanism for the photochemical generation of hydrogen radicals.

## Graphical abstract

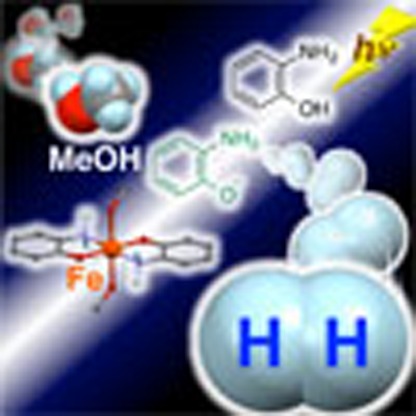

Molecular hydrogen (H_2_) is one of the most promising energy sources of the future^1^,^2^,^3^,^4^,^5^,^6^. As gaseous H_2_ is difficult to handle and store, hydrogen storage materials have been the subject of intensive investigation in recent years^7^,^8^,^9^. Among a number of materials, methanol (MeOH) represents one of the most fascinating hydrogen carriers^10^,^11^,^12^,^13^,^14^,^15^, which is used in fuel cells^16^,^17^,^18^,^19^ due to its high-gravimetric H_2_ content (12.6 wt% H_2_). Since the generation of H_2_ from MeOH is an endothermic reaction, both dehydrogenation catalysts and high temperatures are usually required for this reaction to proceed, and several heterogeneous^20^ and homogeneous^21^,^22^,^23^,^24^,^25^,^26^ catalyst systems have been investigated in this context^27^,^28^. Homogeneous catalytic systems for the dehydrogenation of MeOH can be classified into three types: (1) thermal dehydrogenation catalysts for hydrous MeOH (MeOH reforming), (2) thermal dehydrogenation catalysts for anhydrous MeOH and (3) photochemical dehydrogenation catalysts for anhydrous MeOH. Beller *et al*. reported that the use of [Ru^II^(H)Cl(PNP)] (PNP=HN(C_2_H_4_P*i*-Pr_2_)_2_)^21^ and of the non-precious metal complex [Fe^II^(H)(BH_4_)(PNP)]^22^ allowed a dehydrogenation of the MeOH/H_2_O mixture to CO_2_ (or CO_3_^2−^) at 91 °C. Other examples were reported by Grützmacher and co-workers^23^, and Crabtree and co-workers^29^, who demonstrated that the dehydrogenation of MeOH is catalysed at 91 °C by [Ru^II^(H)(1,4-bis(5H-dibenzo[a,d]cyclohepten-5-yl)-1,4-diazabuta-1,3-diene)]^−^ or [Ir^I^(CO)_2_(*N,N*-dimethylheterocyclic carbene)_2_]^+^ complexes, respectively. Milstein and co-workers^26^ observed the formation of H_2_ from mixtures of MeOH/H_2_O/THF in the presence of [Ru^II^(H)(Cl)(CO)(BPy-^*t*^PNN)] (BPy-^*t*^PNN=6-di-*tert*-butylphosphinomethyl-2,2′-bipyridine) at 60 °C. Conversely, Saito *et al*. and Shinoda *et al*.^30^,^31^,^32^,^33^ reported several Ru-complexes as thermal dehydrogenation catalysts for anhydrous MeOH, which operate at 64–79 °C (TONs_H2_=8–34; TOF_H2_=0.94–1.23 h^−1^).

One promising strategy to lower the undesirably high reaction temperatures are photochemical reactions^34^,^35^,^36^,^37^,^38^,^39^. Photochemical dehydrogenations of anhydrous MeOH should be highly attractive for two reasons: anhydrous HCHO, produced form anhydrous MeOH, is an important intermediate for the production of a large number of industrial chemicals^40^, and the lack of effective catalysts for the removal of H_2_O from aqueous HCHO renders this process relatively cost intensive. To the best of our knowledge, only six homogenous photocatalysts or catalyst precursors for the photochemical dehydrogenation of anhydrous MeOH have been reported during the past three decades (Supplementary Table 1)^34^,^35^,^36^,^37^,^38^,^39^. Saito and co-workers^36^,^37^,^38^,^39^ reported the dehydrogenation of anhydrous MeOH under concomitant formation of H_2_ at 64–65 °C, using Rh, Pd and Ir-based precious metal catalysts. The reaction could also be carried out at 20 °C using *cis*-[Rh^I^_2_(Cl)_2_(CO)_2_(dpm)_2_] (dpm=bis(diphenylphosphino)methane), albeit that the presence of acetone as an additional photosensitizer was required, which led to the formation of considerable amounts of by-products, such as HOC(CH_3_)_2_C(CH_3_)_2_OH^36^. These pioneering studies motivated us to develop a new catalytic platform for the dehydrogenation of anhydrous MeOH at ambient temperature, which requires neither precious metals nor additional photosensitizers.

We have recently reported that [Fe^II^(opda)_3_]^2+^ (opda=*o*-phenylenediamine) is able to photochemically generate H_2_ in tetrahydrofuran^41^. In this reaction, opda does not only act as a chelating ligand via the two amino groups, but also as a multi-electron and proton-pooling site for the photochemical generation of H_2_ at ambient temperature. We proposed that H_2_ is released as a result of the photochemical activation of the N–H bonds in the amino moieties of opda, accompanied by an oxidation to afford the oxidized semi-benzoquinodiimine or *o*-benzoquinodiimine species.

In contrast to the homoleptic coordination of opda via two amino groups, the corresponding *o*-aminophenolato ligand (apH^−^) displays a heteroleptic coordination pattern via one amino and the deprotonated hydroxyl group, which should facilitate the transfer of two electrons and one proton (Fig. 1)^42^,^43^,^44^,^45^,^46^. Pino and co-workers^47^ have reported the ^1^π-π*-initiated photochemical activation of the O–H bond of *o*-aminophenol (apH_2_) in the gas phase. This characteristic reactivity, which occurs on the picosecond timescale in the gas phase at 65–90 °C, has recently been labelled ‘excited state hydrogen detachment (ESHD)'. However, there is a considerable gap between the ESHD of aromatic amines and alcohols and the photochemical hydrogen evolution reaction (PHER) of [Fe^II^(opda)_3_]^2+^, because the generation of H_2_ has not yet been reported for the ESHD system.

Recently, successful examples on the elucidation of electron- and proton-transfer properties of transition metal complexes of apH^−^, together with the characterization of corresponding complexes with *o*-iminosemiquinonate (isq^−^) have been reported, and these studies are currently under extensive investigation as models for non-haem-type iron dioxygenase^42^,^43^,^44^,^45^,^46^. In contrast to these sophisticated examples for the reactivity of apH_2_ derivatives, which proceed under the exclusion of light, examples on photochemical reactions still remain elusive. Here we report the photochemical dehydrogenation of anhydrous MeOH at room temperature catalysed by apH_2_, apH^−^ and the non-precious metal complex *trans*-[Fe^II^(apH)_2_(MeOH)_2_] (**1**). These photocatalysts promote the PHER from MeOH in the absence of additional photosensitizers, and generated H_2_ and HCHO with the highest external quantum yields (*Φ*_H2_) reported so far. Mechanistic investigations revealed that the photo-induced formation of hydrogen radicals triggers the PHERs. Moreover, the complexation between Fe^II^ and apH^−^ facilitates the photochemical generation of H_2_ at longer wavelengths. The observed PHER activity promises potential for these compounds to serve as a new photocatalyst platform.

## Results

### PHERs of MeOH by apH_2_ and apH^−^

Figure 2 shows the ultraviolet–visible–near infrared spectra of apH_2_ (2.00 mM) and apH^−^ (2.00 mM), whereby the latter was generated *in situ* by the treatment of apH_2_ with one equivalent of tetra-*n*-butyl ammonium hydroxide (TBAOH). For both apH_2_ and apH^−^, two absorptions were observed at *λ*_max_ (*ɛ*/M^−1^ cm^−1^)=230 (6,260) and 285 nm (3,020), as well as at 230 (5,540) and 288 nm (2,630), respectively (Supplementary Table 2). These bands were assigned to two π−π* transitions^47^, and the observed similarity of the spectral profiles indicates a negligible effect of the deprotonation of apH_2_ on the π−π* transitions.

MeOH solutions of both apH_2_ and apH^−^ did not exhibit any signs of gas evolution under the exclusion of light. However, the photoirradiation (289±10 nm; 3.2 mW Hg–Xe lamp) of a MeOH solution of apH_2_ induced the evolution of gas, and a gas chromatography (GC) analysis of a gaseous sample taken from the headspace of the reaction vessel allowed the detection of H_2_ (Supplementary Fig. 1). After 5 h of photoirradiation, 4.1±0.21 equiv. of H_2_ per molecule of apH_2_ (*Φ*_H2_=2.9±0.15%) were obtained (Fig. 3, Table 1, and Supplementary Fig. 2), while up to 6.5±0.33 equiv. H_2_ per molecule of apH_2_ were obtained after 24 h of photoirradiation (Supplementary Fig. 3), suggesting that the reaction is not stoichiometric, but catalytic. Deprotonated apH^−^ exhibits a similar PHER activity (*Φ*_H2_=3.7±0.19%) on irradiation at 289±10 nm. Control reactions, using neat MeOH under otherwise identical conditions, did not exhibit any PHER activity. Considering these PHER results and the aforementioned absorption spectra, it is feasible to assume that the π−π* excitation of apH_2_ and apH^−^ initiates the PHERs.

In general, oxidation of anhydrous MeOH should afford oxidized species such as formaldehyde (HCHO)^37^,^38^,^39^ and methyl formate (HCO_2_Me)^31^,^33^. In the case that small amount of H_2_O contaminates, formic acid (HCO_2_H)^37^,^38^,^39^ and CO_2_^21^,^22^ are also produced as minor products. After PHER of apH_2_ (5 h), a high-performance liquid chromatography (HPLC) analysis revealed that 3.3±0.08 equiv. of HCHO were generated per molecule of apH_2_ (Table 1 and Supplementary Fig. 4a). Taking the experimental errors (3 standard deviation (s.d.)) into account, the difference between the quantities of the two products is statistically not significant, corroborating the photochemical dehydrogenation of MeOH. Furthermore, prolonged PHER (24 h) afforded a small amount of HCO_2_H and/or HCO_2_Me (HCO_2_H/HCO_2_Me), which was produced from dehydrogenation of HCHO in the presence of trace amounts of residual H_2_O, in addition to H_2_ and HCHO (Table 1 and Supplementary Fig. 4b). This reaction thus represents the first example of an organophotocatalyst promoting the photochemical dehydrogenation of MeOH to generate H_2_. In particular, the external quantum yields (apH_2_: *Φ*_H2_=2.9±0.15; apH^−^: *Φ*_H2_=3.7±0.19%) are the highest value in those of previously reported molecular photocatalysts (for more details, see Supplementary Table 1)^25^,^30^,^31^,^32^.

### PHERs of MeOH by *trans*-[Fe^II^(apH)_2_(MeOH)_2_] (1)

**Figure 1:**
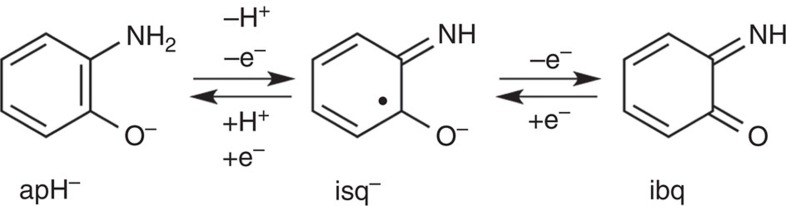
Electron- and proton-transfer properties of apH^−^. On removal of one proton and one-electron, *o*-aminophenolate (apH^−^) reversibly affords *o*-iminosemiquinonate (isq^−^), which is reversibly transferred into *o*-iminobenzoquinone (ibq) on a further one-electron oxidation.

Mixing two equivalents of apH_2_ and TBAOH with Fe^II^(ClO_4_)_2_·6H_2_O in MeOH under an atmosphere of N_2_ afforded colourless crystals of **1** (Supplementary Table 3). The molecular structure of **1** is shown in Fig. 4, and selected bond distances are summarized in the Supplementary Table 4. The structure of **1** is characterized by a pseudo-octahedral coordination geometry of the Fe atom, which is located on an inversion centre, and by two bidentate NO ligands that occupy the equatorial positions, while two monodentate O ligands reside on the apical positions. The two chelating ligands adopt a *trans* geometry, most likely to reduce steric repulsions^48^. The assignment of the two chelating ligands requires great care, as these could be apH^−^, isq^−^ or *o*-iminobenzoquinone (ibq) ligands (Fig. 1)^42^,^43^,^44^,^45^,^46^. In complex **1**, the observed C–N and C–O bond distances of 1.461(5) Å and 1.342(5) Å, respectively, suggest a single-bond character for these bonds^41^,^43^,^44^,^45^,^46^,^49^. Moreover, the observed C–C bond distances of 1.385–1.417(6) Å within the six-membered ring indicate high levels of aromaticity. While apH_2_ contains one hydroxyl and two amino protons, which can be deprotonated by TBAOH (1 equiv.) to give monoanionic C_6_H_4_NOH_2_^−^, previous reports on Fe(II) complexes of apH^−^ have shown that it is possible to deprotonate the hydroxyl proton in apH_2_ selectively^43^,^44^,^45^,^46^. As the Fe–O1 (2.048(3) Å) and Fe–N1 (2.214(4) Å) bond distances in **1** are comparable to those in these examples (Fe–O: 1.931–1.994(2) Å; Fe–N: 2.212–2.299(7) Å), the structure of the chelating ligands should be commensurate with that of apH^−^. Furthermore, the selective deprotonation of the hydroxyl proton was consistent with typical p*K*_a_ values for aromatic amines (for example, p*K*_a_ aniline=30.6)^50^ and alcohols (for example, p*K*_a_ phenol=18.0)^51^. While the observed Fe–O2 bond distance (2.229(3) Å) is comparable to those of typical Fe^II^–HOMe bonds (2.205(16) Å)^52^, it is substantially longer than typical Fe^II^–OMe bonds (1.782(3) Å)^53^. Accordingly, the axial ligands should be MeOH ligands. Moreover, the Fe–N1, Fe–O2 and Fe–O1 bond distances of 2.214(4) Å, 2.229(3) Å and 2.048(3) Å, respectively, suggest the presence of a high-spin Fe^II^ centre^43^,^44^,^45^,^46^. These observations, in their entirety, suggest that **1** adopts a structure that is consistent with *trans*-[Fe^II^(apH)_2_(MeOH)_2_].

It should be noted that the Fe–N1 bond distance (2.214(4) Å) in **1** is similar to those in [Fe^II^(opda)_3_](ClO_4_)_2_ (2.204–2.234(2) Å), whereas the dihedral angle between the O1–Fe1–N1 and O1–C1–C2–N1 planes in **1** (1.06°) is relatively small compared with those in [Fe^II^(opda)_3_](ClO_4_)_2_ (35.90, 38.17 and 44.15°)^41^. The high levels of planarity in the five-membered rings that contain the Fe^II^ centre in **1** are indicative for the presence of interactions between the *d*-orbitals of Fe^II^ and the π-orbitals of the apH^−^ moieties via the *p*-orbitals on the O atom(s)^43^. It is also noteworthy that the observed differences with respect to the dihedral angles induce a substantially different coordination environment for the amino groups in **1** relative to those in [Fe^II^(opda)_3_](ClO_4_)_2_ (ref. 41).

**Figure 2:**
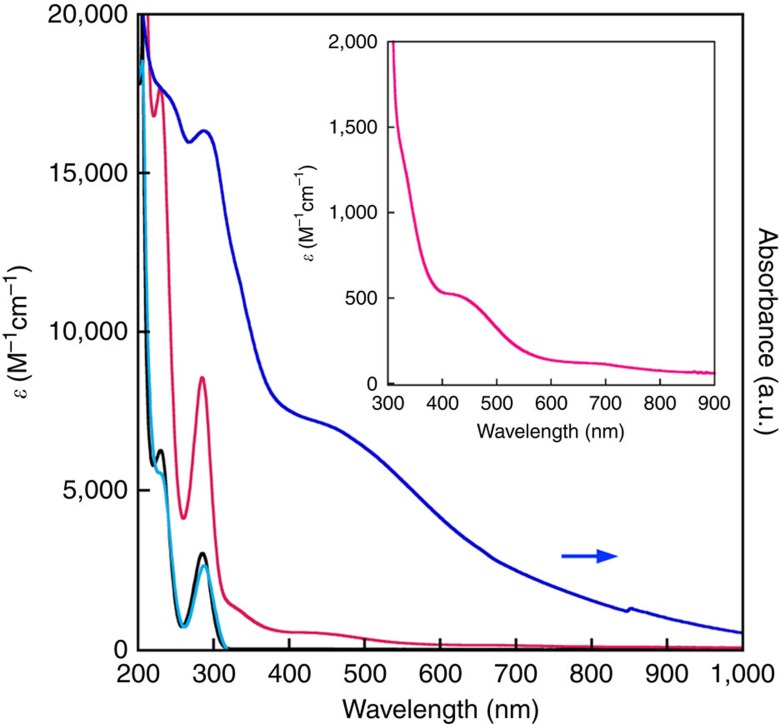
Ultraviolet–visible–near infrared spectra of apH_2_-based photocatalysts in MeOH. [apH_2_ (black line)]=[apH^−^ (turquoise line)]=2 mM; [**1** (magenta line)]=1.35 mM. The generation of apH^−^ was accomplished *in situ* by the treatment of apH_2_ with TBAOH. The solid-state spectrum of **1** (KBr disk, blue line) is shown for comparison. The inset shows a magnification for the 300–900 nm region of the solution spectrum of **1**.

The ultraviolet–visible–near infrared spectra of **1** (1.35 mM) in MeOH, as well as that of solid **1** (KBr disk) are shown in Fig. 2. For **1**, four absorption bands were observed at *λ*_max_ (*ɛ*/(M^−1^ cm^−1^))=230 (17,690), 285 (8,550), 335 (1,210), 460 (480) and 680 nm (118). The two absorption bands at 230 and 285 nm, with relatively large molar extinction coefficients, are similar to those of apH_2_ and apH^−^, and were therefore assigned to the transitions involving apH^−^-centred π−π* transitions^47^. The two bands at 335 nm and 460 nm, which were not observed for apH_2_ and apH^−^, were tentatively assigned to charge transfer (CT) transitions between Fe(II) and the apH^−^ ligands^45^, while the *d–d* transitions of the Fe(II) centre appeared as a shoulder band at 680 nm (118 M^−1^ cm^−1^). In the solid state, the absorption bands of **1** appeared at 235, 287, 335, 450 and 750 nm, similar to those of the MeOH solution, suggesting comparable structures in solution and in the solid state. As metal ions are known to interact with apH^−^ and perturb its properties, the bis-(*o*-aminophenolato) Cu(II) analogue [Cu^II^(apH)_2_(H_2_O)] was synthesized and structurally characterized to assess possible effects of the presence of metal ions (see Supplementary Note 1, as well as Supplementary Figs 5 and 6, and Supplementary Table 2). Under an atmosphere of N_2_ and under the exclusion of light, **1** is reasonably stable in MeOH (Supplementary Fig. 7a). In contrast, the corresponding Cu(II) complex exhibited a time-dependent spectral change under the same conditions, which is indicative of the formation of 2-aminophenoxazine-3-one (APX) (Supplementary Figs 7b and 8). These results suggest a remarkable influence of the metal centre on the electron-donating ability of the apH^−^ ligand. As the Cu complex proved to be unstable in MeOH, no PHER experiments were carried out on this complex.

**Figure 3:**
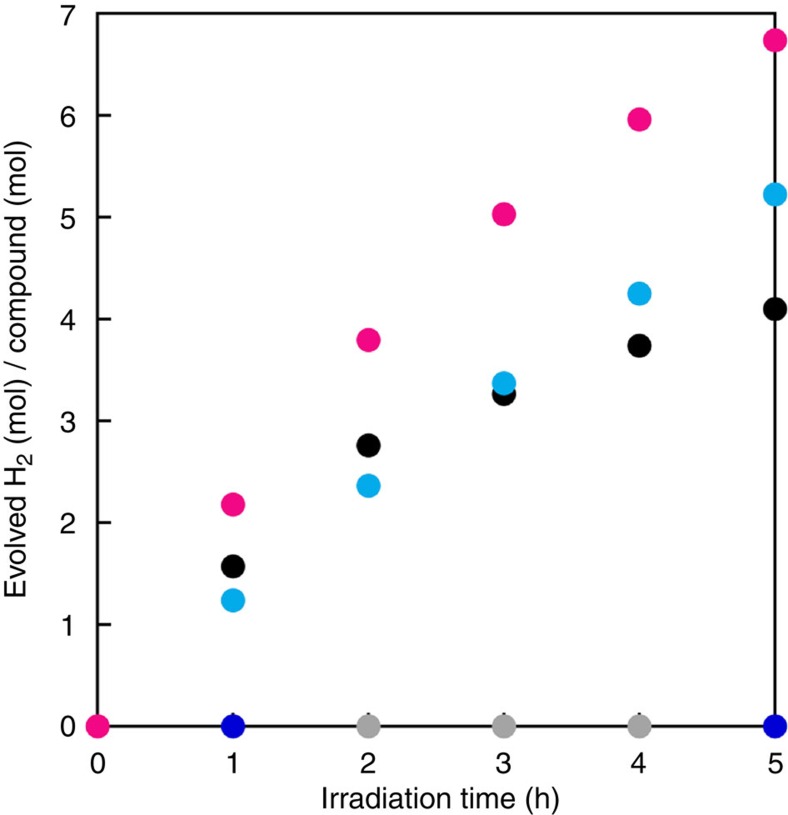
H_2_ evolution from MeOH solutions of apH_2_-based photocatalysts. Mols of evolved H_2_ per mols of catalyst as a function of PHER time for MeOH solutions of apH_2_ (2 mM; black circles), apH^−^ (2 mM; turquoise circles), **1** (1 mM; magenta circles), and Fe^II^(ClO_4_)_2_·6H_2_O (1 mM; blue circles), together with pure MeOH (grey circles). Irradiation wavelength, 289±10 nm (3.2 mW). Estimated relative s.d., 5%.

**Figure 4:**
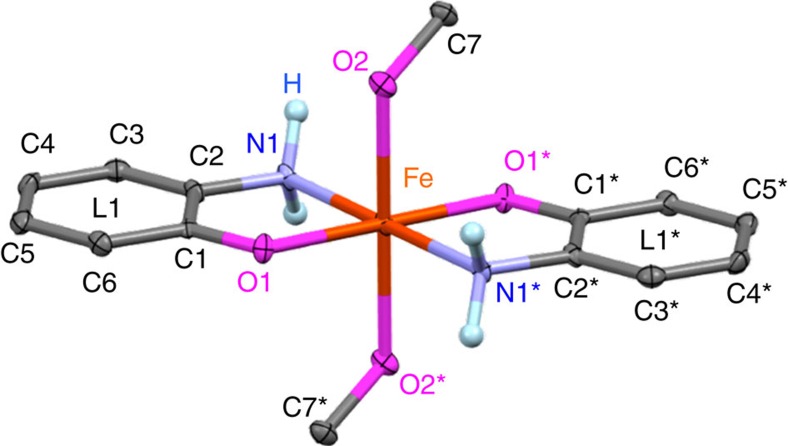
Molecular structure of 1. Atomic displacement parameters set at 50% probability; colour code: C, dark grey; Fe, orange; N, light blue; O, magenta; ball-and-stick plots for N-bound hydrogen atoms (light blue), while all other hydrogen atoms are omitted for clarity. C1/C1*, C2/C2*, C3/C3*, C4/C4*, C5/C5*, C6/C6*, C7/C7*, N1/N1*, N2/N2*, O1/O1* and O2/O2* denote pairs of crystallographically equivalent atoms related by the symmetry operation 2–*x*, –*y*, 2–*z*.

Figure 3 shows the amount of evolved H_2_ as the result of the photoirradiation (*λ*_irr_=289±10 nm; 3.2 mW Hg–Xe lamp; 5 h) of a MeOH solution of **1** (magenta circles). The MeOH solution of **1** also showed PHER activity, resulting in the formation of 6.7±0.34 (5 h) and 14.9±0.75 (24 h) mols of H_2_ per mol of **1**, respectively (Supplementary Fig. 3). In addition to H_2_ and HCHO, prolonged PHER (24 h) also afforded small amounts of HCO_2_H/HCO_2_Me (Table 1), while evidence for the formation of CO_2_ was not observed. Control reactions under the same conditions, using a MeOH solution of Fe^II^(ClO_4_)_2_·6H_2_O did not show any PHER activity (Fig. 3 and Supplementary Fig. 2). Moreover, a MeOH solution of Fe^II^(ClO_4_)_2_·6H_2_O containing 2 equiv. of opda generated only minor amounts of H_2_, indicating a lower activity of the opda complex of Fe(II) relative to that of **1** (Supplementary Fig. 9). For the PHER of a MeOH solution of **1**, a *Φ*_H2_ value of 4.8±0.24% (5 h) was estimated. Considering these PHER results and the aforementioned absorption spectra, it is feasible to suggest that the photoexcitation of the apH^−^ ligand in **1** initiates the PHER. Even when the PHER of **1** was carried out at 45 °C, no temperature dependence was observed, which in turn suggests that the photochemical process should be the rate-determining step of the PHER (Supplementary Fig. 10). Interestingly, the photocatalytic activity on apH^−^ was not suppressed even after the complexation with the Fe^II^ centre, implying the possibility to tune the reactivity of the system via a variation of the metal ion.

**Figure 5:**
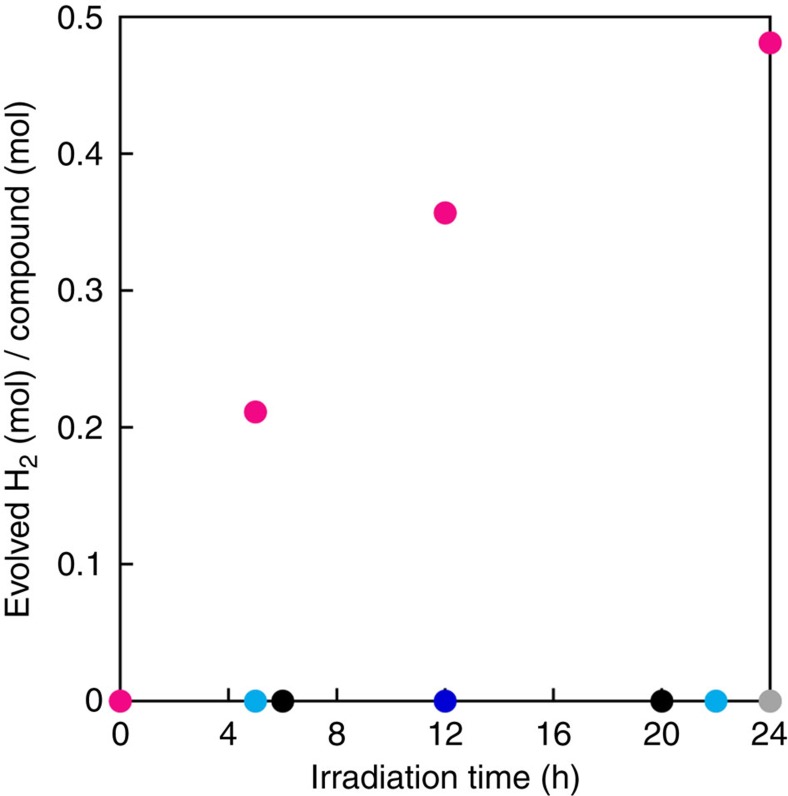
H_2_ evolution from a MeOH solution of 1. Mols of evolved H_2_ per mol of catalyst as a function of PHER time for MeOH solutions of apH_2_ (2 mM; black circles), apH^−^ (2 mM; turquoise circles), **1** (1 mM; magenta circles) and Fe^II^(ClO_4_)_2_·6H_2_O (1 mM; blue circles), together with pure MeOH (grey circles). Irradiation wavelength, 350±10 nm (21.8 mW). Estimated relative s.d., 5%.

To determine the excitation that initiates the PHER of **1** in MeOH, we examined the correlation between the PHER performance and the wavelength of the irradiation source. Exposing a MeOH solution of **1** to photoirradiation at 460±10 nm (*ɛ*_460 nm_=483 M^−1^ cm^−1^, 24 h) did not generate any H_2_. However, exposing a MeOH solution of **1** to photoirradiation at 350±10 nm (21.8 mW, *ɛ*_350 nm_=932 M^−1^ cm^−1^, 24 h) initiated PHER and resulted in the formation of 0.48±0.02 equiv. of H_2_ with an estimated *Φ*_H2_ value of 0.019±0.001% (Fig. 5 and Supplementary Fig. 11). Although the *Φ*_H2_ value for an irradiation at 350±10 nm (0.019±0.001%) is lower than that at 289±10 nm (4.8±0.24%), these results imply that the PHER may be driven by lower-energy light sources, given a suitable combination of apH^−^-type ligands and metal ions.

### A reaction mechanism for the PHERs

**Figure 6:**
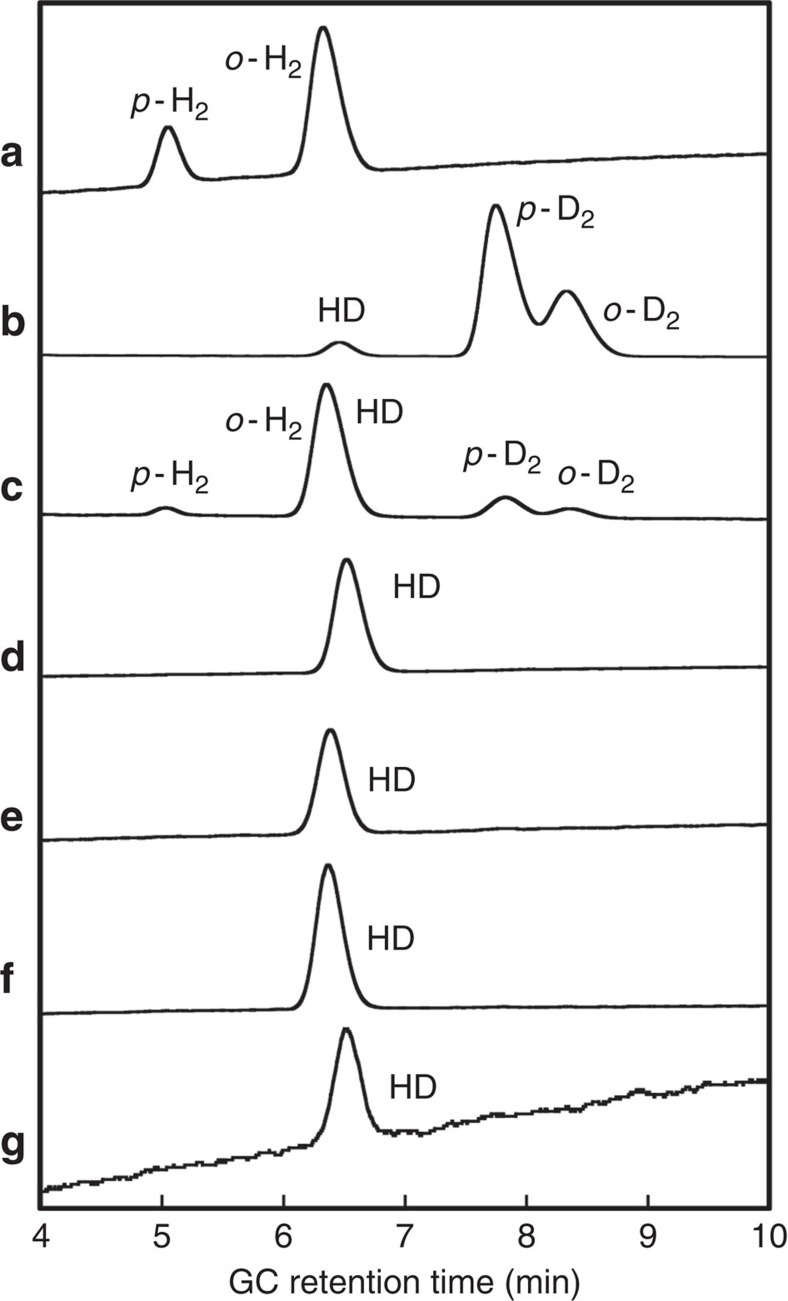
GC analysis of the gaseous PHER products. GC profiles of (**a**) H_2_, (**b**) D_2_ and HD generated by treatment of MeOH-*d*_4_ (99.8%) with sodium metal, and (**c**) HD, H_2_ and D_2_ generated by the treatment of a mixed MeOH/MeOH-*d*_4_ solution (*v*/*v*=1/1) with sodium metal; gas samples obtained from MeOH-*d*_3_ (99.5%) solutions of (**d**) apH_2_, (**e**) apH^−^ and (**f**) **1** after photoirradiation at 289±10 nm (3.2 mW, 24 h) and (**g**) **1** after photoirradiation at 350±10 nm (21.8 mW, 24 h); GC conditions: 8% KOH alumina column; *T*=77 K.

For the photochemical dehydrogenation of anhydrous MeOH, reports on detailed investigations regarding the mechanism on a molecular level remain scarce so far^35^,^36^,^37^,^38^,^39^. Therefore, we wanted to shed some light on the fundamental PHER mechanism that is operative in the MeOH solutions of apH_2_, apH^−^ and **1**, and we were especially interested in the source of the evolved H_2_. In this system, it is pertinent to distinguish proton and electron sources according to: (i) the hydroxyl and methyl protons of MeOH, (ii) the aromatic protons of apH_2_, apH^−^ and apH^−^ in **1**, and (iii) the amino protons of apH_2_, apH^−^ and apH^−^ in **1**. Taking the amount of photochemically generated H_2_ and HCHO into account (Table 1), together with the small amounts of HCO_2_H/HCO_2_Me, it seems plausible to consider (i) as the most probable source of the evolved H_2_. To experimentally confirm this hypothesis, PHERs were carried out in MeOH-*d*_3_, and the evolved gas was analysed by GC at 77 K (Fig. 6). For H_2_, D_2_ and HD standards, retention times of 5.0 (*p*-H_2_), 6.3 (*o*-H_2_), 7.8 (*p*-D_2_), 8.4 (*o*-D_2_) and 6.4 min (HD) were observed (Fig. 6a–c). Photoirradiation of apH_2_ in MeOH-*d*_3_ at 289±10 nm afforded gas samples that exhibited a single peak with a retention time of 6.4 min. (Fig. 6d), suggesting the selective formation of HD under such PHER conditions. Clearly, the D atom in the evolved HD originates from an *α*-hydrogen of MeOH-*d*_3_, thus suggesting a C–H(D) bond cleavage during PHER. The PHER of apH^−^ and **1** (Fig. 6e,f), as well as the photoirradiation of a MeOH-*d*_3_ solution of **1** at 350±10 nm (Fig. 6g) furnished samples that displayed similar HD peaks in the GC analysis. These results confirm that the PHER of MeOH solutions of apH_2_, apH^−^ and **1** generate H_2_ from MeOH.

As apH_2_ and apH^−^ can promote one-proton and two-electron transfers, it is not surprising that these can generate electron(s), hydrogen radical(s) or a hydride (Fig. 1). To gain a better mechanistic understanding of the observed PHERs, photochemical reactions were carried out in the presence of 2-methylpropane-2-thiol (*t*-BuSH), which is able to act as a hydrogen radical scavenger on account of the relatively low-bond dissociation energy of the S–H bond, resulting in the formation of di-*tert*-butyl disulfide (*t*-Bu_2_S_2_)^54^.

**Figure 7:**
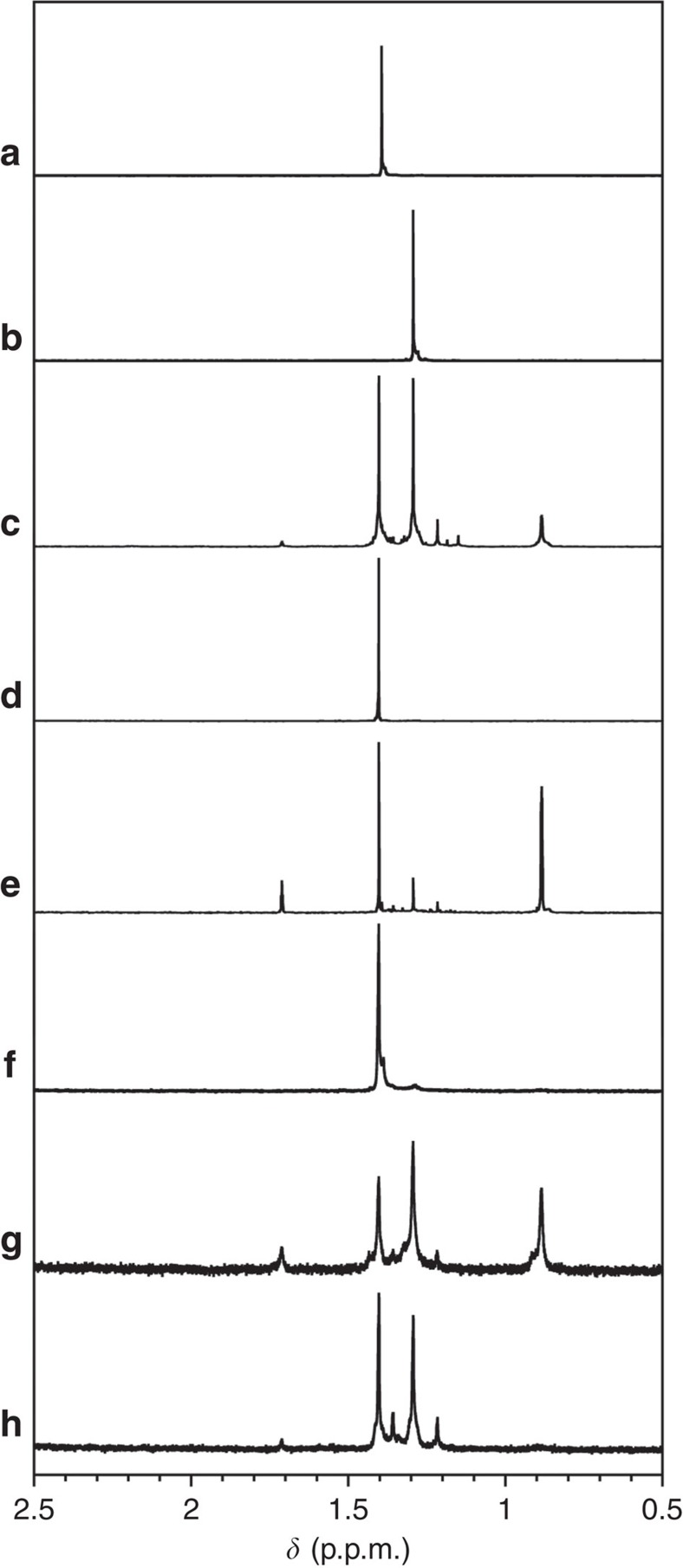
^1^H NMR spectra of PHER products in the presence of hydrogen radical scavengers. ^1^H NMR spectra (MeOH-*d*_4_) of (**a**) *t*-BuSH, (**b**) *t*-Bu_2_S_2_, (**c**) *t*-Bu_2_S_2_ after photoirradiation at 289±10 nm (5 h), (**d**) *t*-BuSH with apH_2_, (**e**) *t*-BuSH with apH_2_ after photoirradiation at 289±10 nm (5 h), (**f**) *t*-BuSH with **1**, (**g**) *t*-BuSH with **1** after photoirradiation at 289±10 nm (5 h) and (**h**) *t*-BuSH with **1** after photoirradiation at 350±10 nm (24 h).

The ^1^H NMR spectrum of *t*-BuSH prior to photoirradiation at 289±10 nm (24 h) in MeOH-*d*_4_ is shown in Fig. 7a. A singlet peak was observed at *δ*=1.40 p.p.m. and assigned to the protons of the *t*-Bu group. After photoirradiation, no considerable change was observed in the spectrum (Supplementary Fig. 12), which is consistent with the absence of an absorption ∼289 nm (Supplementary Fig. 13). The ^1^H NMR and ultraviolet–visible spectra of *t*-Bu_2_S_2_ in MeOH-*d*_4_ and MeOH are shown in Fig. 7b and Supplementary Fig. 13, respectively. In MeOH-*d*_4_, the ^1^H NMR spectrum of *t*-Bu_2_S_2_ exhibits a singlet resonance at 1.29 p.p.m., while the ultraviolet–visible spectrum shows an absorption ∼289 nm in MeOH (Supplementary Fig. 13). After photoirradiation of a MeOH-*d*_4_ solution of *t*-Bu_2_S_2_ at 289±10 nm (5 h), major singlet peaks emerged at 1.71, 1.40, 1.29 and 0.88 p.p.m. together with several minor peaks, which demonstrates the photochemical reactivity of *t*-Bu_2_S_2_ (Fig. 7c). These resonances are thus indicative of the *in situ* formation of *t*-Bu_2_S_2_ and the reaction products from its photochemical decomposition.

The ^1^H NMR spectrum of a mixture of *t*-BuSH and apH_2_ in MeOH-*d*_4_ (Fig. 7d and Supplementary Fig. 14) displays a peak at 1.40 p.p.m., which is comparable to the resonances of pure *t*-BuSH in MeOH-*d*_4_ (Fig. 7a), thus suggesting negligible interaction between apH_2_ and *t*-BuSH in the ground state. After photoirradiation of this solution at 289±10 nm (5 h), new singlet peaks emerged at 1.71, 1.29, 1.22 and 0.88 p.p.m. (Fig. 7e). The new peaks are identical to those obtained for photoreacted *t*-Bu_2_S_2_ (Fig. 7c), suggesting the formation of *t*-Bu_2_S_2_ during the photoreaction of apH_2_ and *t*-BuSH.

Similar experiments were also carried out for **1**, but the ^1^H NMR spectrum of *t*-BuSH in the presence of **1** (Fig. 7a,f, and Supplementary Fig. 15) did not indicate any significant interaction between *t*-BuSH and paramagnetic **1** in the ground state. After photoirradiation of this solution at 289±10 nm (5 h), new singlet peaks were observed at 1.71, 1.29, 1.22 and 0.88 p.p.m. (Fig. 7g). These peaks are comparable to those of the photoirradiation product obtained from *t*-BuSH and apH_2_ (Fig. 7e), and these results thus suggest the formation of *t*-Bu_2_S_2_ from *t*-BuSH in the presence of **1**.

At this point, it should be beneficial to consider possible formation mechanisms for the formation of *t*-Bu_2_S_2_ from *t*-BuSH. One possibility is the oxidation of *t*-BuSH by apH_2_ in the excited state, while another is the generation of hydrogen radicals from photoirradiated apH_2_, followed by the abstraction of a hydrogen radical from *t*-BuSH. On excitation at 285 nm in MeOH, apH_2_ exhibits an emission peak at 342 nm (Supplementary Fig. 16a). This emission was tentatively assigned to the ^1^π–π* excited state, which was not quenched by *t*-BuSH. Therefore, the generation of a hydrogen radical from an alternative excited path represents a more likely mechanism compared with an electron transfer via the ^1^π–π* excited state. Similar to the case of apH_2_, the emission peak of **1** in MeOH was not influenced by the presence of *t*-BuSH on excitation at 285 nm (Supplementary Fig. 16b). This result suggests that the PHER of **1** does not include the oxidation of *t*-BuSH by the excited state of **1**, but the generation of a hydrogen radical from **1**.

Furthermore, the characteristic singlet peak for *t*-Bu_2_S_2_ at 1.29 p.p.m. was observed after photoirradiation of **1** at 350±10 nm (24 h; Fig. 7h). These results suggest that the pathway for the generation of hydrogen radials is also included in the excitation of **1** at 350±10 nm, similarly to the excitation of **1** and apH_2_ at 289±10 nm. The photoirradiation of **1** in MeOH at 350±10 nm in the presence of 3-carbamyl-1-methylpyridinium chloride (NADCl), which is an electron or hydride scavenger^55^,^56^, revealed no considerable change in the ^1^H NMR spectra prior and posterior to the photoreaction (Supplementary Fig. 17). These results indicate that the photoreaction of **1** at 350±10 nm does not include any electron transfer or generation of hydrides, and these are thus consistent with a mechanism based on hydrogen radicals (for a plausible reaction mechanism in the presence of *t*-BuSH, see Supplementary Fig. 18).

In general, hydrogen can be generated from MeOH using an electron donor, a hydrogen radical or a hydride. Reactions between MeOH-*d*_3_ and sodium metal (electron donor) or NaBH_4_ (hydride donor) showed that under these conditions, H_2_ was produced selectively (equations 1 and 2, Fig. 6a–c, and Supplementary Fig. 19). In contrast, the formation of HD was negligible, as the *o*-H_2_/*p*-H_2_ peak area ratio was almost identical to that of the H_2_ standard (Supplementary Table 5). Hydrogen radicals have previously been reported to react with the *α*-hydrogen atoms of MeOH to produce H_2_ and the corresponding ^·^CH_2_OH radical (equation 3)^57^. The ^·^CH_2_OH radical is a good reducing agent and able to react with electron acceptors (EA) such as Fe(III) or Co(III), which affords HCHO as the oxidized species (equation 4).


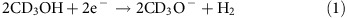



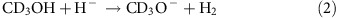



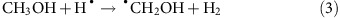



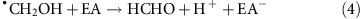


For apH_2_, proton- and electron-transfer properties should be expected, and apH_2_ should thus be able to donate electrons, hydrides and/or hydrogen radicals. The ability to photochemically generate hydrogen radicals from the hydroxyl protons has already been reported for apH_2_ (ref. 47). As previously mentioned, we confirmed that PHERs of MeOH-*d*_3_ solutions of apH_2_, apH^−^ and **1** selectively generate HD (Fig. 6), which suggests a selective abstraction of the *α*-hydrogen atoms from MeOH. Actually, the generation of hydrogen radicals was confirmed in the photochemical reactions of apH_2_ and **1** in the presence of the scavengers (Fig. 7). Consequently, all PHERs should be initiated by the generation of a hydrogen radical.

**Figure 8:**
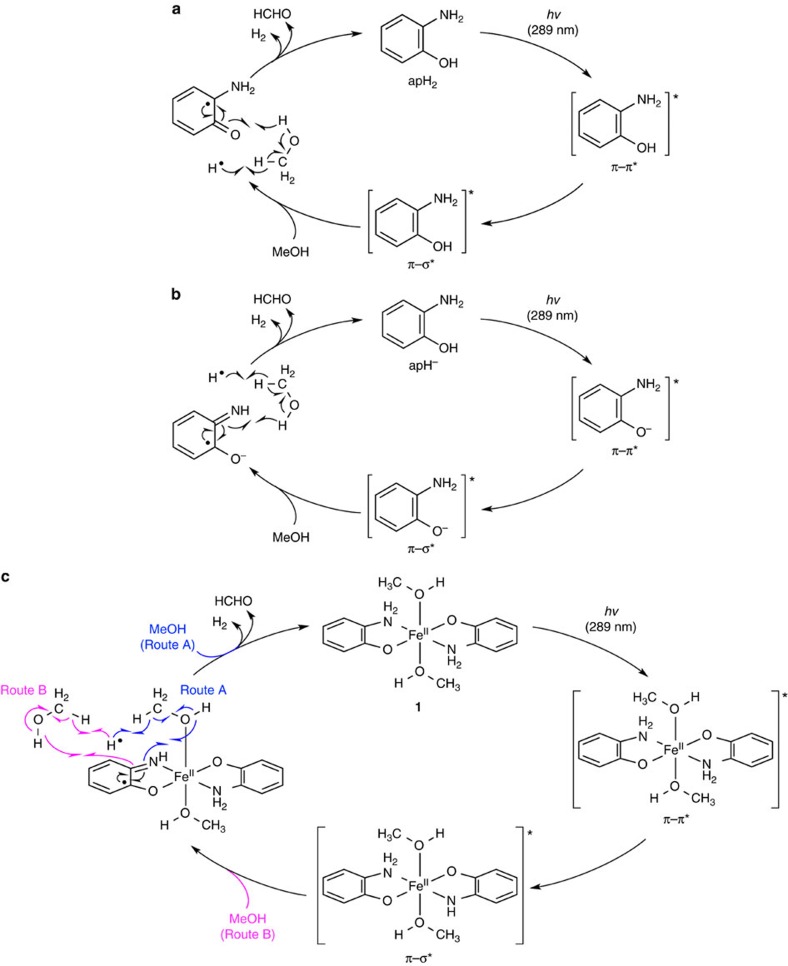
Plausible PHER mechanisms. Reaction cycles catalysed by (**a**) apH_2_, (**b**) apH^−^ and (**c**) **1** upon irradiation at 289±10 nm.

Figure 8a shows a plausible mechanism for the PHER involving apH_2_. It seems reasonable to assume that PHER (289±10 nm) proceeds via the initial photochemical generation of hydrogen radicals from the hydroxyl moieties of apH_2_. Subsequently, H_2_ and HCHO should be formed by the selective abstraction of *α*-hydrogen atoms from MeOH (Fig. 6). The additional oxidation products (HCO_2_H/HCO_2_Me) would then be generated from the dehydrogenation of HCHO or from the dehydrogenation of HCHO in the presence of residual trace amounts of water.

In the case of apH^−^, a hydroxyl proton is not present, and therefore, O–H bond cleavage in apH^−^ should not occur during PHER. Previous studies on aromatic amines, such as aniline and opda, revealed characteristic photoreactions, which are initiated by the π−π* excitation through the 3*s* Rydberg states of the nitrogen atom in the amino group^58^,^59^,^60^. Eventually, the N–H *σ*-bonds in these amino moieties are photochemically activated to generate hydrogen radicals via the π−π*/π−σ* conical intersection in these reactions, similar to the case of apH_2_ (refs 61, 62). The formation of a hydrogen radical and isq^−^ may be possible from the homolytic cleavage of an N–H bond in the π−σ* exited state from a π−π* excitation (Fig. 8b), and subsequent reactions indicate the generation of H_2_ and HCHO, similar to the case of apH_2_.

Furthermore, complex **1**, containing apH^−^ ligands, should also generate hydrogen radicals from the homolysis of an N–H bond in the apH^−^ moiety under photoirradiation conditions (289±10 nm; Fig. 8c). In the plausible mechanism for **1**, the MeOH molecule coordinated to the Fe(II) centre and/or that not directly bound to the Fe(II) centre may be included in the reaction as described in routes A and B (Fig. 8c). Remarkably, the complexation of apH^−^ with Fe(II) does not inhibit its PHER activity. It should also be noted here that the PHER proceeding via excitations including π−π* transitions show higher *Φ*_H2_ values (4.8±0.24%) relative to CT excitations (0.019±0.001%).

## Discussion

In this paper, we report the first examples for the photocatalytic dehydrogenation of anhydrous MeOH at room temperature, using apH_2_, apH^−^ and an Fe(II) complex of apH^−^ (**1**) as photocatalysts. These photocatalysts promote the PHER from MeOH in the absence of additional photosensitizers, and generate hydrogen and formaldehyde. For these PHERs, *Φ*_H2_ values based on the amount of generated H_2_, HCHO and HCO_2_H/HCO_2_Me were estimated using defined excitations at 289±10 or 350±10 nm. The observed PHER activity and the comparable *Φ*_H2_ values of apH_2_ and apH^−^ promise potential for these compounds as a new organophotocatalyst platform. Furthermore, **1** demonstrated a comparable photochemical reactivity and *Φ*_H2_ value relative to apH^−^, despite of the presence of a paramagnetic Fe(II) centre. The PHER activity on photoexcitation of the CT band of **1** suggested that the complexation between Fe^II^ and apH^−^ allows access to unprecedented photoreactivity that is able to realize the photochemical generation of H_2_ at longer wavelengths compared with apH_2_ and apH^−^. The central issues to be addressed in the immediate future concern improvements of the catalytic activity and the use of visible light as a driving force. These topics are currently under investigation in our laboratory, using various combinations of metal ions with apH^−^-type ligands and extended π-systems.

## Methods

### General procedures

Unless noted otherwise, all synthetic operations and measurements were carried out under an atmosphere of N_2_ using Schlenk-line techniques. Fe^II^(ClO_4_)_2_·6H_2_O, Cu^II^(OAc)_2_·H_2_O (OAc=acetate), HCO_2_H, acetic acid (AcOH), ammonium acetate (NH_4_OAc), acetyl acetone, calcium oxide (CaO) and H_2_SO_4_ were purchased from Wako Pure Chemical Industries (Japan). Dehydrated MeOH, dichloromethane (CH_2_Cl_2_) and MeOH-*d*_4_ were purchased from Kanto Chemical Co. Inc. (Japan). TBAOH in MeOH (37%), which was used after the removal of MeOH, an aqueous solution of HCHO (37%), *t*-BuSH, *t*-Bu_2_S_2_, NADCl and apH_2_ were purchased from Tokyo Chemical Industry Co. Ltd. (Japan). Prior to use, apH_2_ was washed with CH_2_Cl_2_ and dried *in vacuo* for several minutes, while *t*-BuSH was used after stirring with CaO for 12 h, followed by distillation. MeOH-*d*_3_ was purchased from Sigma-Aldrich. APX was prepared according to a reported procedure^63^. All solvents that were used under anaerobic conditions were thoroughly degassed by at least five freeze–pump–thaw cycles immediately prior to use. Although we did not experience any difficulties with perchlorate salts, these should be regarded as potentially explosive, and therefore handled with utmost care.

### *trans*-[Fe^II^(apH)_2_(MeOH)_2_] (1)

A colourless MeOH solution (5 ml) of apH_2_ (144 mg, 1.31 mmol) and TBAOH (340 mg, 1.31 mmol) was slowly deposited onto an aqua-blue MeOH (5 ml) solution of Fe^II^(ClO_4_)_2_·6H_2_O (240 mg, 0.661 mmol) under an atmosphere of N_2_. After leaving the solution to stand at 3 °C for a period of 5 days, colourless crystals of **1**, suitable for X-ray crystallographic analysis, were obtained (see also Supplementary Methods and Supplementary Data 1). Crystalline **1** was isolated by filtration, washed with MeOH (2 × 4 ml), and dried *in vacuo* (yield: 59%). Crystals of **1** were found to be highly hygroscopic, and always contained small amounts of water. Anal. Calc. for C_14_H_20.4_FeN_2_O_4.2_ (**1**+0.2 H_2_O): C, 49.49; H, 6.05; N, 8.24. Found: C, 49.49; H, 6.08; N, 8.27.

### *trans*-[Cu^II^(apH)_2_(H_2_O)] (2)

A colourless MeOH solution (10 ml) of apH_2_ (200 mg, 1.83 mmol) was added to 20 ml of a blue aqueous solution of Cu^II^(OAc)_2_·H_2_O (184 mg, 0.92 mmol), resulting in the formation of an aqua-blue suspension after stirring the reaction mixture for 5 min. Complex **2** was isolated as an aqua-blue powder by filtration, washed with MeOH (3 × 3 ml) and Et_2_O (2 × 3 ml), before being dried *in vacuo* (yield: 83%). Anal. Calc. for C_12_H_14_CuN_2_O_3_ (**2**): C, 48.40; H, 4.74; N, 9.41. Found: C, 48.13; H, 4.75; N, 9.17. Single crystals, suitable for X-ray crystallographic analysis, were obtained by layering an aqueous solution of apH_2_ onto an aqueous solution of Cu^II^(OAc)_2_·H_2_O (see also Supplementary Methods and Supplementary Data 2).

### Equipment for the PHERs

A 200 W Hg–Xe lamp (LC8, Hamamatsu Photonics) with a quartz light guide (*Φ*5 L9588) was used as the light source. For photoirradiation at 289±10 nm, a 289 nm band pass filter (BPF) was used, while a 350 nm BPF (03 type filter) was used for irradiation at 350±10 nm. For photoirradiation at 460±10 nm, a 100 W Xe lamp (LAX-103, Asahi Spectra Co., Ltd.) with a quartz light guide (*Φ*5 × 1,000L UD0030), a VISIBLE-type mirror module and a 460 nm BPF were used. The intensity of the light was measured using a power meter (NOVA, Ophir optronics Ltd.) and a thermopile sensor (3A, Ophir optronics Ltd.). The released quantities of H_2_, HD and D_2_ were measured using a gas chromatograph (GC, Shimadzu GC-2014), equipped with a 2 m column packed with either MS 5A (*T*=343 K; carrier gas: Ar) or 8% KOH alumina (*T*=77 K; carrier gas: He).

### PHER by apH_2_ in MeOH

A handmade Schlenk-flask-equipped quartz vessel (*V*=115 ml) was charged with 1 ml of a methanolic apH_2_ solution that was prepared by dissolving apH_2_ (4.36 mg, 0.04 mmol) in MeOH (20 ml). Subsequently, the solution was exposed to photoirradiation in a water bath at room temperature. Gas samples (0.3 ml) were collected from the headspace of the vessel using a gas-tight syringe (Tokyo Garasu Kikai Co. Ltd.) and analysed by GC (MS 5A column). Estimated relative s.d. for H_2_ (mol)/apH_2_ (mol) and *Φ*_H2_: 5%.

### PHER by apH^−^ in MeOH

The quartz vessel was charged with 1 ml of a methanolic apH^−^ solution that was prepared by dissolving apH_2_ (4.36 mg, 0.04 mmol) and TBAOH (10.4 mg, 0.04 mmol) in MeOH (20 ml). Subsequently, the solution was exposed to photoirradiation in a water bath at room temperature. Gas samples (0.3 ml) were collected from the headspace of the vessel using a gas-tight syringe and analysed by GC, similar to the aforementioned procedure. Estimated relative s.d. for H_2_ (mol)/apH^−^ (mol) and *Φ*_H2_: 5%.

### PHER by 1 in MeOH

Crystals of **1** were filtered and washed with MeOH (2 × 4 ml), before being dissolved in MeOH (4 ml) to afford a saturated pale-orange MeOH solution of **1**. Subsequently, 1 ml of this solution was transferred into the quartz vessel. The reaction and analysis were carried out as described above. Estimated relative s.d. for H_2_ (mol)/**1** (mol) and *Φ*_H2_: 5%.

### Analysis of HCHO in PHER solutions

An aqueous solution of AcOH (100 ml, 2.26 M) was prepared by dissolving 12.9 ml of AcOH (226 mmol) in water under an atmosphere of air. Subsequently, an aqueous solution of NH_4_OAc (200 ml, 2.25 M) was prepared by dissolving NH_4_OAc (34.68 g, 450 mmol) in water (200 ml). An AcOH/NH_4_OAc buffer solution was prepared by combining 8 ml of the aqueous AcOH solution with 200 ml of the aqueous NH_4_OAc solution. An aqueous solution (1,000 ml) of acetyl acetone and ethanol was prepared by dissolving 7 ml of acetyl acetone and 14 ml of ethanol in water (979 ml). Methanolic standard solutions of HCHO (0, 1, 5 and 10 mM) were prepared by diluting a methanolic HCHO solution (37%) with appropriate amounts of dehydrated MeOH. A mixture of the methanolic standard solutions of HCHO (0.1 ml), the AcOH/NH_4_OAc buffer solution (2.0 ml) and the aqueous acetyl acetone solution (2.0 ml) was heated to 60 °C for 15 min. Then, the solution was cooled to room temperature, and 20 μl of the reaction mixture were analysed by HPLC, using a Synergi 4u Hydro-RP80A column (Phenomenex) and H_2_O/MeCN (*v*/*v*=85/15) at a flow rate of 1.0 ml min^−1^. The targeted 3,5-diacetyl-1,4-dihydro-2,6-lutidine, which was generated from HCHO, was detected at 370 nm, and the apparent linearity of the thus obtained calibration curves suggested that a quantitative analysis of the compound in the PHER solutions should be possible (Supplementary Fig. 4a). To estimate the amount of HCHO produced in the PHER solutions, 0.1 ml of the corresponding reaction mixtures were used instead of the standard solutions. Estimated relative s.d. based on the calibration curves for the PHER products of HCHO: 1.4–2.5% (refs 64, 65, 66, 67, 68).

### Analysis of HCO_2_H/HCO_2_Me in the PHER solutions

Methanolic standard solutions of HCO_2_H (0, 0.5, 1, 2 and 4 mM) were prepared by diluting formic acid with MeOH in water under an atmosphere of air. About 500 μl of each solution were treated with 5 μl of H_2_SO_4_ at room temperature and stirred for 15 min. These HCO_2_H standard solutions were analysed by HPLC using a Synergi 4u Hydro-RP80A column and H_2_O/MeCN (*v*/*v*=95/5) at a flow rate of 1.0 ml min^−1^. Quantities of HCO_2_H/HCO_2_Me (methyl formate) were calculated based on the detected HCO_2_Me at 210 nm; given the apparent linearity of the calibration curves, a quantitative analysis of HCO_2_H/HCO_2_Me in the PHER solutions should be possible (Supplementary Fig. 4b). To estimate the total amount of HCO_2_H/HCO_2_Me produced in the PHER solutions, 500 μl of the corresponding reaction mixtures were used instead of standard solutions. Estimated relative s.d. based on the calibration curves for the PHER products of HCO_2_H/HCO_2_Me: 1.9–2.9% (refs 67, 68). During the PHERs, HCO_2_Me may either be generated directly from MeOH, or it may be generated from the methyl-esterification of HCO_2_H with H_2_SO_4_ in the presence of MeOH (HPLC pre-treatment). During the PHERs, HCO_2_H may be produced from the dehydrogenation of HCHO in the presence of trace amounts of residual H_2_O, especially in the case of apH^−^, which was generated *in situ* from the deprotonation of apH_2_ by TBAOH.

### PHER by 1 in MeOH-*d*_3_

All reactions and analyses were carried out as described above, except that **1** was dissolved in MeOH-*d*_3_, and that an 8% KOH alumina column was used for the GC analysis. The H_2_ standards were detected at 5.0 (*p*-H_2_) and 6.3 min (*o*-H_2_) (Fig. 6a). D_2_ gas was generated from the reaction of MeOH-*d*_4_ with sodium metal, and the corresponding peaks were detected at 7.8 (*p*-D_2_) and 8.4 min (*o*-D_2_) (Fig. 6b). HD gas was generated from the reaction of a MeOH/MeOH-*d*_4_ (*v*/*v*=1/1) mixture with sodium metal, and the corresponding signals for HD were detected at 6.4 min (Fig. 6c). Since pure HD gas was unobtainable, the calibration and quantitative estimation of generated HD could not be carried out.

### PHER by apH_2_ and 1 in the presence of scavengers

All reactions were carried out as described above for the PHER procedures, except for using a quartz NMR tube as the reaction vessel and MeOH-*d*_4_ as the solvent. For that purpose, apH_2_ or **1** were dissolved in degassed MeOH-*d*_4_ (apH_2_: 2 mM; **1**: 1 mM) with *t*-BuSH or NADCl (2 mM), before the solutions were analysed by ^1^H NMR spectroscopy prior and posterior to photochemical reactions.

### Physical measurements

^1^H NMR (500 MHz) spectra were measured on a JEOL EX-500 spectrometer. Elemental analyses were carried out on a Perkin-Elmer 2400 II CHN analyzer. Ultraviolet–visible–near infrared spectra (200–3,300 nm) in solution or in the solid state (KBr pellets) were recorded on a Hitachi U-4100 spectrophotometer at 296 K. HPLC measurements were carried out on a Shimadzu LC-20AT liquid chromatograph, equipped with an SPD-20A ultraviolet–visible detector. Emission spectra were recorded on a Horiba fluoromax using MeOH solutions of apH_2_ and **1** at 296 K.

### Calculations of external quantum yields (*Φ*_H2_)

MeOH solutions of apH_2_, apH^−^ and **1** were irradiated at 289±10 or 350±10 nm. The amount of H_2_ evolved during the subsequent 5 h was used to calculate the *Φ*_H2_ according to the following equations:


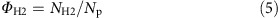



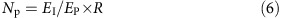



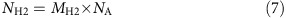



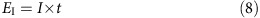



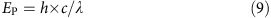



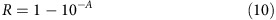


wherein, *N*_H2_, *N*_p_, *E*_I_, *E*_p_, *R*, *M*_H2_, *N*_A_*, I*, *t*, *h*, *c* and *A* refer to the number of molecules of evolved H_2_ per mol of catalyst, the number of absorbed photons, the energy of the irradiation source, the energy of the irradiation photons, the proportion of the catalyst that is absorbing light, the mol number of the evolved H_2_, the Avogadro constant, the optical intensity of the light, the irradiation time, the Planck constant, the speed of light in vacuum and the absorbance of the catalyst in MeOH, respectively. The absorbance throughout the photoreaction was assumed to be constant at an optical length of 1.0 cm: apH_2_ (5.72 at 289 nm), apH^−^ (5.39 at 289 nm) and **1** (7.76 at 289 nm, 1.15 at 350 nm).

### Data availability

The data supporting the results of this study are available from the article and its Supplementary Information file, or from the authors upon request. The X-ray crystallographic coordinates used for the structure determination reported in this article have been deposited at the Cambridge Crystallographic Data Centre (CCDC) under deposition numbers CCDC-1062112 (**1**) and CCDC-1418535 (**2**). These data can be obtained free of charge from The Cambridge Crystallographic Data Centre via www.ccdc.cam.ac.uk/data_request/cif.

## Additional information

**How to cite this article:** Wakizaka, M. *et al*. Dehydrogenation of anhydrous methanol at room temperature by *o*-aminophenol-based photocatalysts. *Nat. Commun.* 7:12333 doi: 10.1038/ncomms12333 (2016).

## Supplementary Material

Supplementary Information Supplementary Figures 1-19, Supplementary Tables 1-5, Supplementary Note 1, Supplementary Methods and Supplementary References

Supplementary Data 1 Crystallographic Information File for complex **1**

Supplementary Data 2 Crystallographic Information File for complex **2**

## Figures and Tables

**Table 1:** PHER products from the photochemical reactions of apH_2_-based photocatalysts*.

Compound	*λ*_irr_ (nm)	*t*_irr_ (h)	Mols per mol of catalyst			*Φ*_H2_ (%)
			H_2_	HCHO†	HCO_2_H/HCO_2_Me‡	
apH_2_	289±10	5	4.1§	3.3§	ND	2.9||
apH^−^	289±10	5	5.2§	4.0§	ND	3.7||
**1**	289±10	5	6.7§	6.7§	ND	4.8||
apH_2_	289±10	24	6.5§	4.9§	0.71§	
apH^−^	289±10	24	14.1§	14.2§	0.73§	
**1**	289±10	24	14.9§	9.9§	0.97§	
apH_2_	350±10	24	ND	ND	ND	
apH^−^	350±10	24	ND	ND	ND	
**1**	350±10	24	0.48¶	1.1¶	ND	0.019||

ND, not detected.

^*^[apH_2_]=[apH^−^]=2 mM; [**1**]=1 mM.

^†^Detected as 3,5-diacetyl-1,4-dihydro-2,6-lutidine.

^‡^Detected as methyl formate.

^§^Estimated relative standard deviation (s.d.) for H_2_ (5%), HCHO (1.4-2.5%) and HCHO/HCO_2_Me (1.9-2.9%) per eq.

^||^External quantum yields were estimated based on the amount of evolved H_2_ after *t*_irr_=5 h, under consideration of a relative s.d. of 5%.

^¶^Possibly associated with an increased experimental error, due to the small amount of product generated.
