# Supplementary material for: Dehydrogenation of anhydrous methanol at room temperature by o-aminophenol-based photocatalysts
Source: Nat Commun. 2016 Jul 26;7:12333. doi: 10.1038/ncomms12333 (PMC4963534; doi:10.1038/ncomms12333)
Supplement: Supplementary Information — Supplementary Figures 1-19, Supplementary Tables 1-5, Supplementary Note 1, Supplementary Methods and Supplementary References [file ncomms12333-s1.pdf]

Supplementary Information

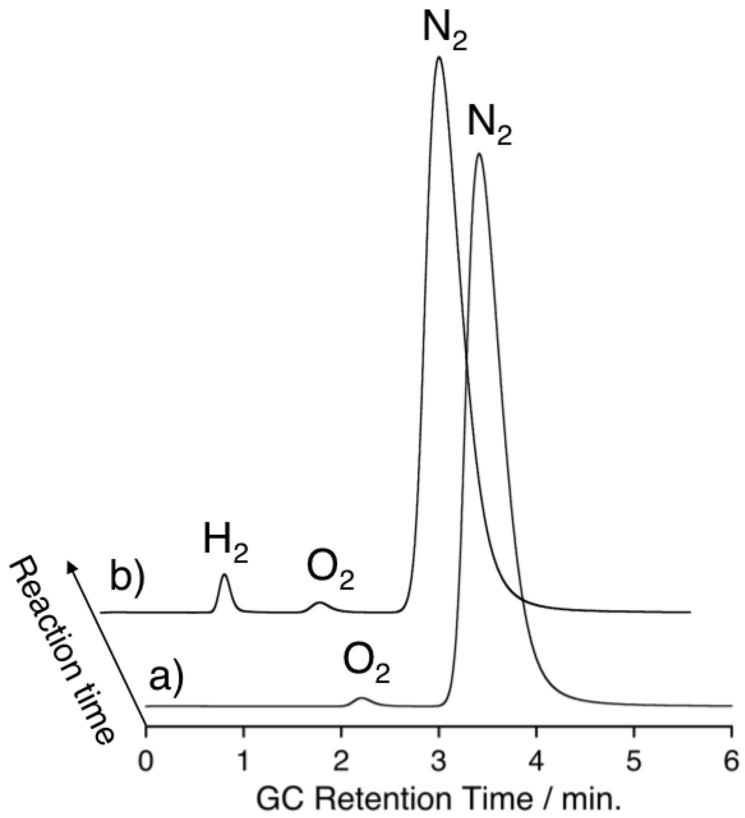

Supplementary Figure 1 | Gas chromatograms for PHER from MeOH solution of apH$_2$. GC analysis for PHERs (289 ± 10 nm; 3.2 mW) of apH$_2$ in MeOH (2 mM) after (**a**) 0 h and (**b**) 5 h.

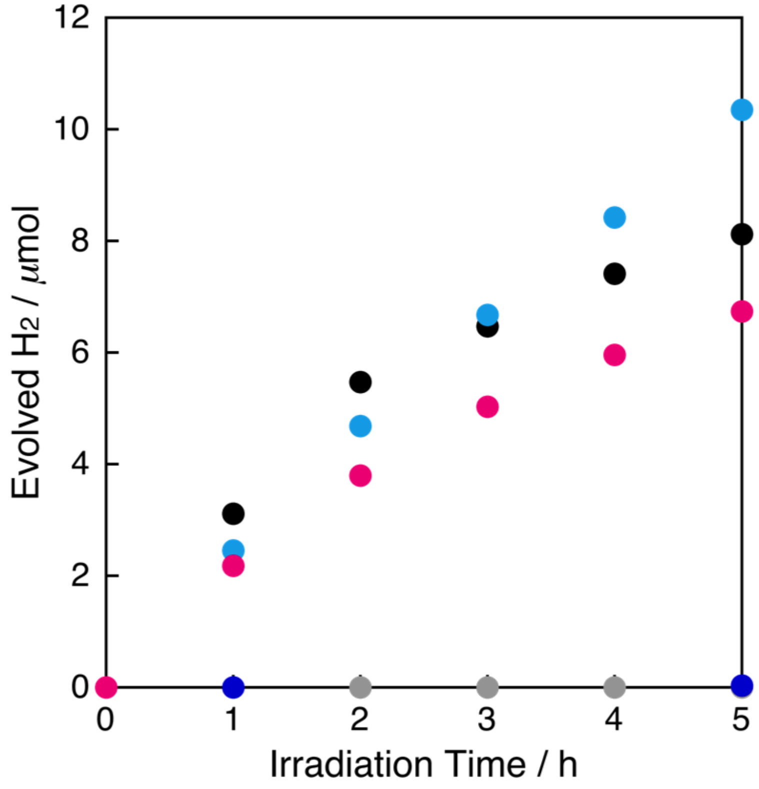

Supplementary Figure 2 | H_2 evolution from MeOH solutions of apH_2-based photocatalysts. Evolution of H_2 (μmol) as a function of PHER time (289 ± 10 nm, 3.2 mW) of MeOH solutions of apH_2 (2 mM; black circles), apH^- (2 mM; turquoise circles), **1** (1 mM; magenta circles), and $Fe^{II}(ClO_4)_2 \cdot 6H_2O$ (1 mM; blue circles), together with pure MeOH (gray circles). Estimated relative standard deviation: 5%.

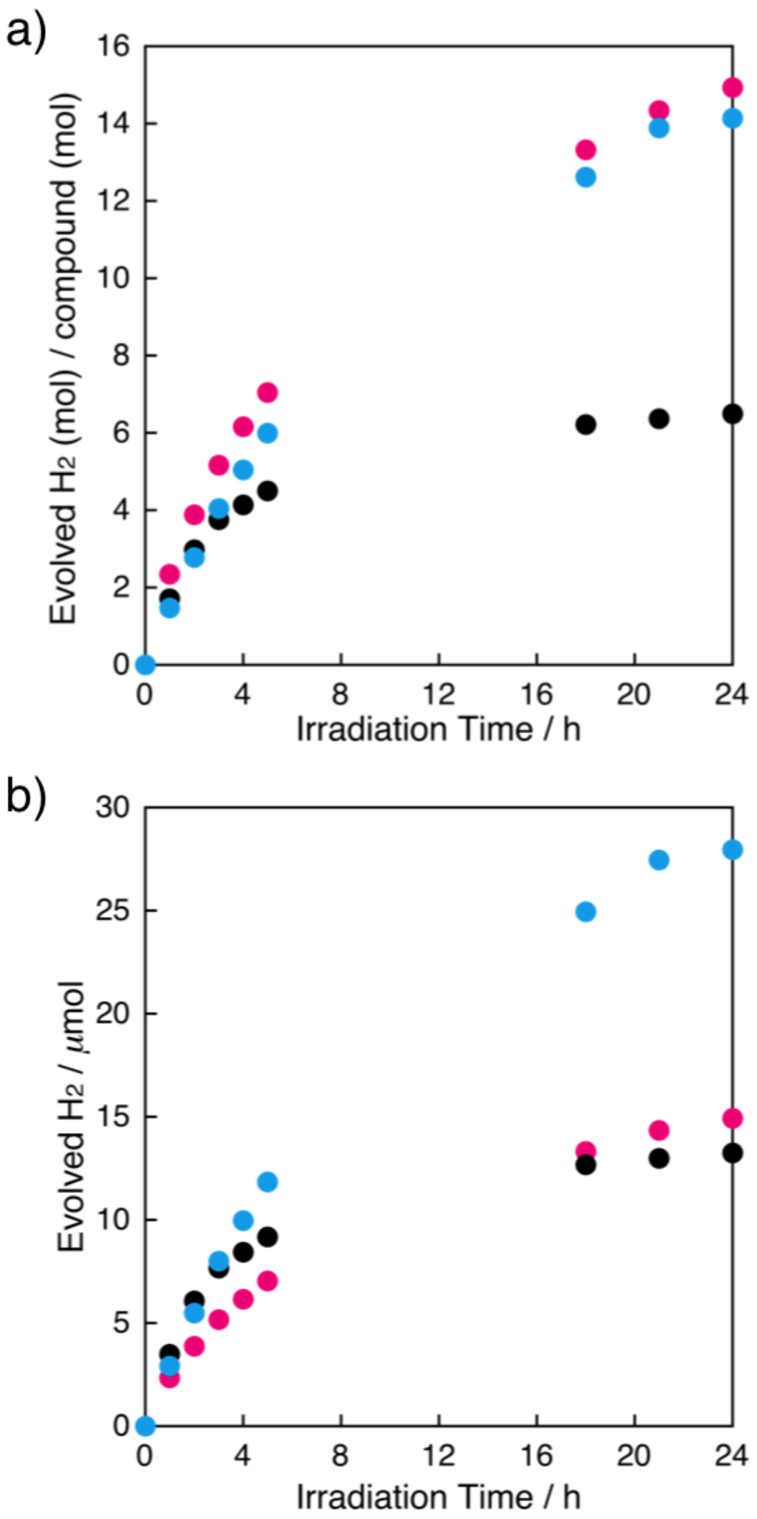

Supplementary Figure 3 | H_2 evolution from MeOH solutions of apH$_2$-based photocatalysts. Mols of evolved of a) H_2 (mol) per mol of catalyst (mol), and b) H_2 (μmol) as a function of PHER time (289 ± 10 nm; 3.2 mW) of MeOH solutions of apH$_2$ (2 mM; black circles), apH$^-$ (2 mM; turquoise circles), and **1** (1 mM; magenta circles). Estimated relative standard deviation: 5%.

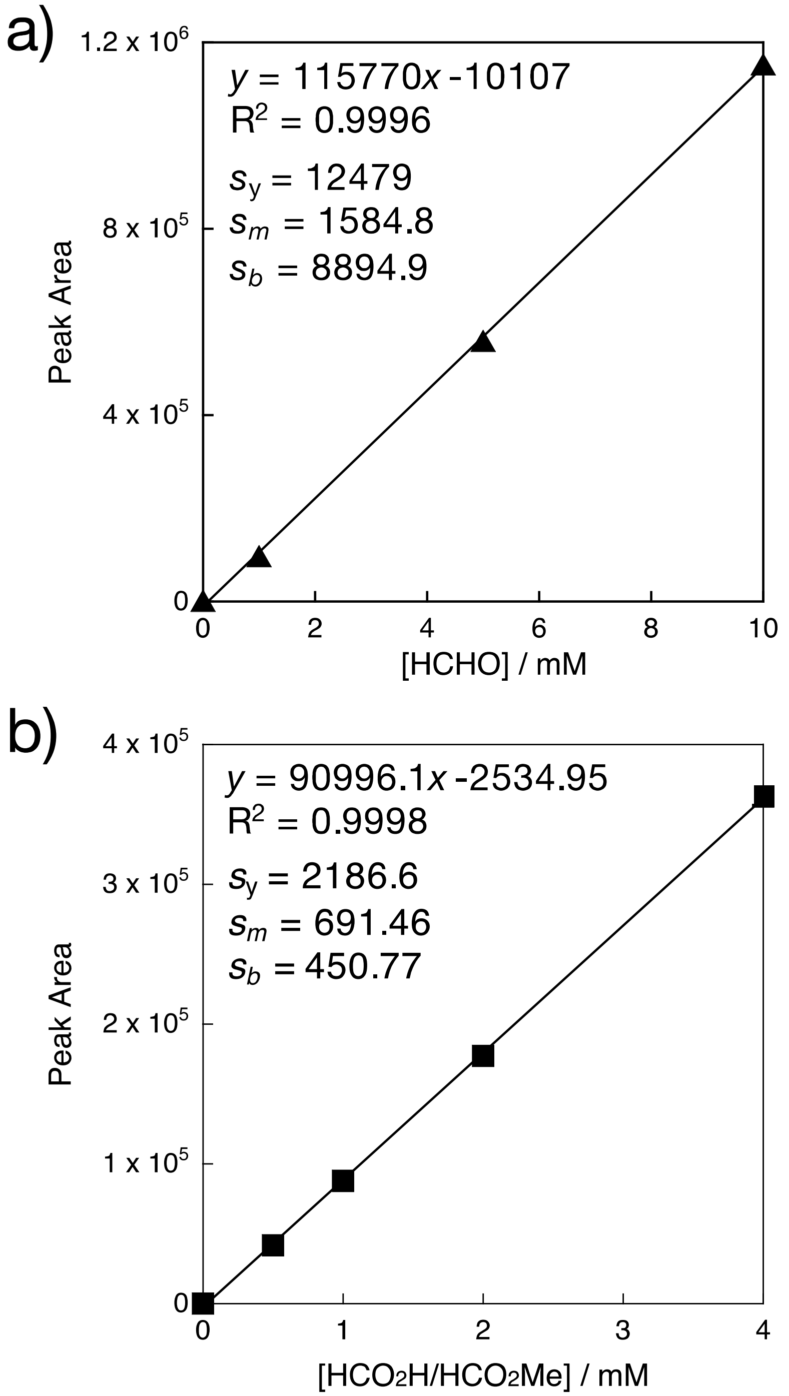

Supplementary Figure 4 | Calibration curves for HCHO and HCO_2H. HPLC calibration curves for (**a**) HCHO and (**b**) HCO_2H/HCO_2Me. The s_y, s_m, and s_b refer to the standard deviations of the regression, slope, and intercept, respectively.

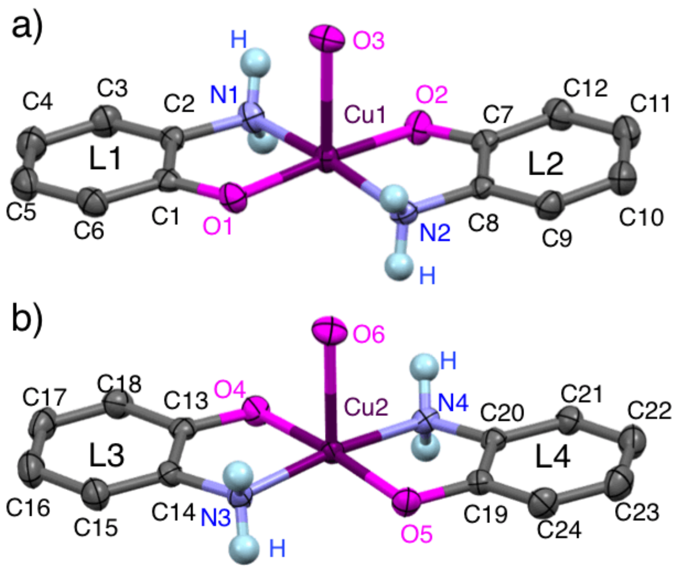

Supplementary Figure 5 | Molecular structure of 2. Molecular structures of the crystallographically independent units (**a**) A and (**b**) B in **2** with atomic displacement parameters set at 50% probability; color code: Cu = purple, O = magenta, N = light blue, and C = dark gray; ball-and-stick plots for N-bound hydrogen atoms (light blue), while all other hydrogen atoms are omitted for clarity.

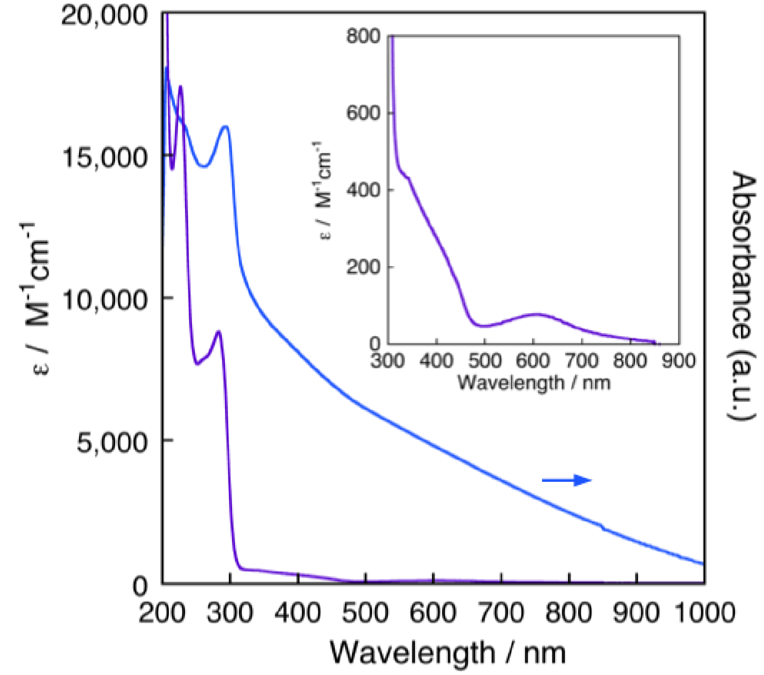

Supplementary Figure 6 | UV-vis-NIR spectra of 2. UV-vis-NIR spectra of **2** in MeOH (1.00 mM; purple line) and in the solid state (KBr disk; blue line) under an atmosphere of N_2. The inset shows a magnification of the 300-900 nm region for the solution spectrum.

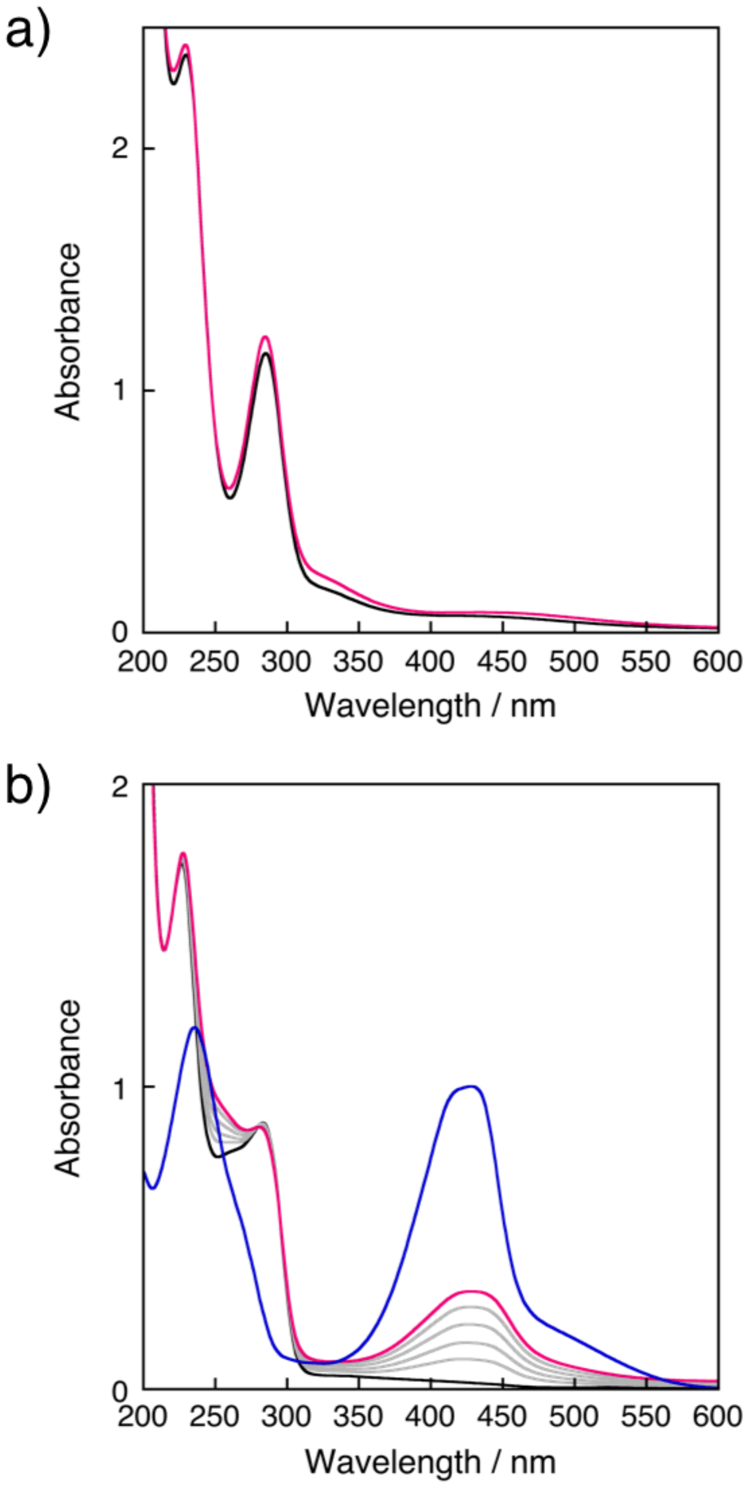

Supplementary Figure 7 | Time course UV-vis-NIR spectra of 1 and 2. UV-vis spectra of (**a**) **1** (0 h; black line) and **1** (20 h; magenta line) (1.35 mM), as well as of (**b**) **2** (0 h; black line), **2** (4, 8, 12, and 16 h; gray lines), and **2** (20 h; magenta line) (1.00 mM) in MeOH under an atmosphere of N_2. The UV-vis spectrum of APX (1 mM; blue line) is shown for comparison.

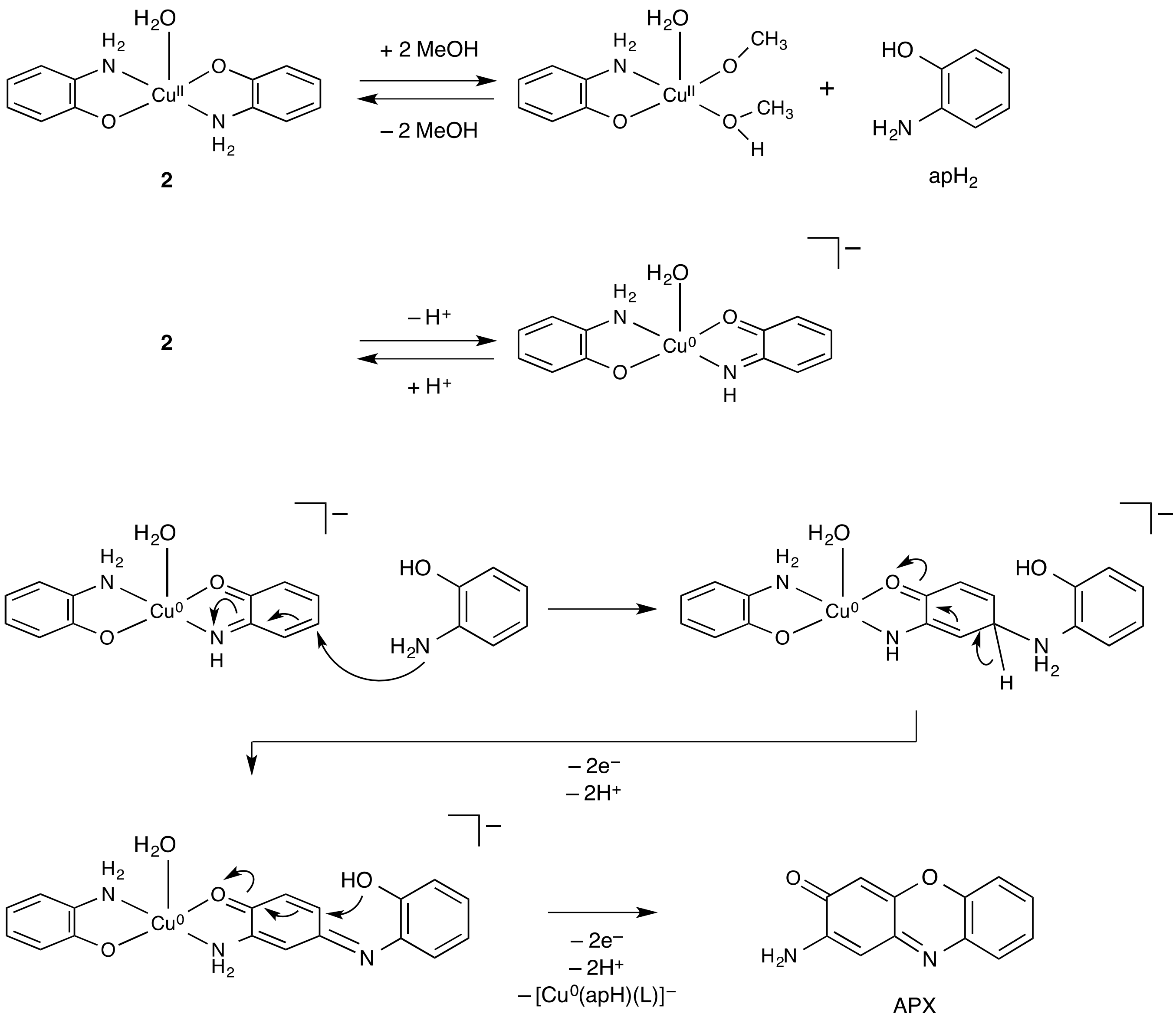

Supplementary Figure 8 | Plausible mechanism. A Plausible mechanism for the formation of APX from **2**.[1]

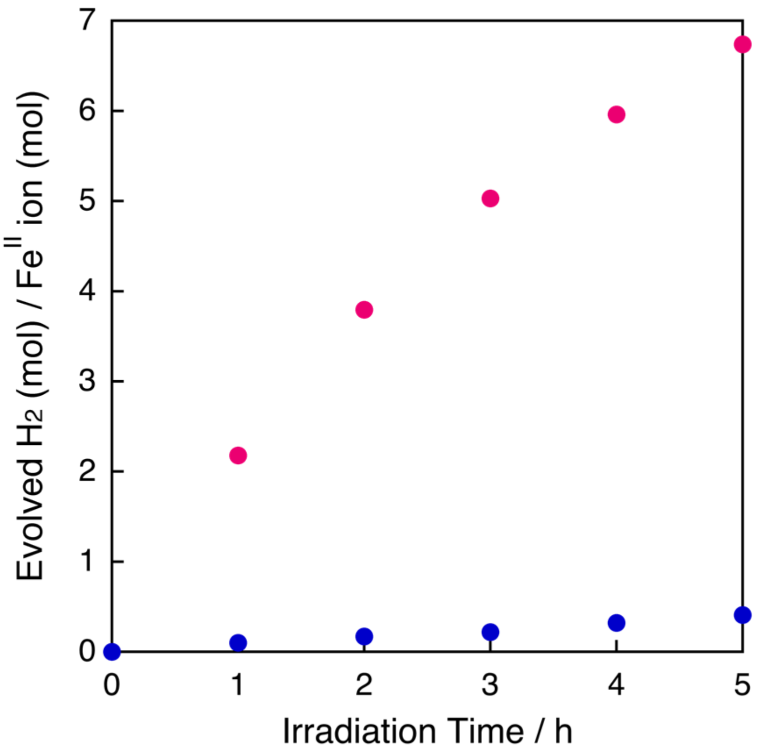

Supplementary Figure 9 | H_2 evolution from MeOH solutions of 1 and opda analogue. H_2 evolution from MeOH solutions of **1** and $Fe^{II}(ClO_4)_2{\cdot}6H_2O$ + 2 opda (289 ± 10 nm, 3.2 mW). Mols of evolved H_2 per mol of Fe^{II} ions as a function of PHER time for MeOH solutions of **1** (1 mM; magenta circles), and $Fe^{II}(ClO_4)_2{\cdot}6H_2O$ + 2 opda (1 mM in Fe(II); blue circles). Estimated relative standard deviation: 5%.

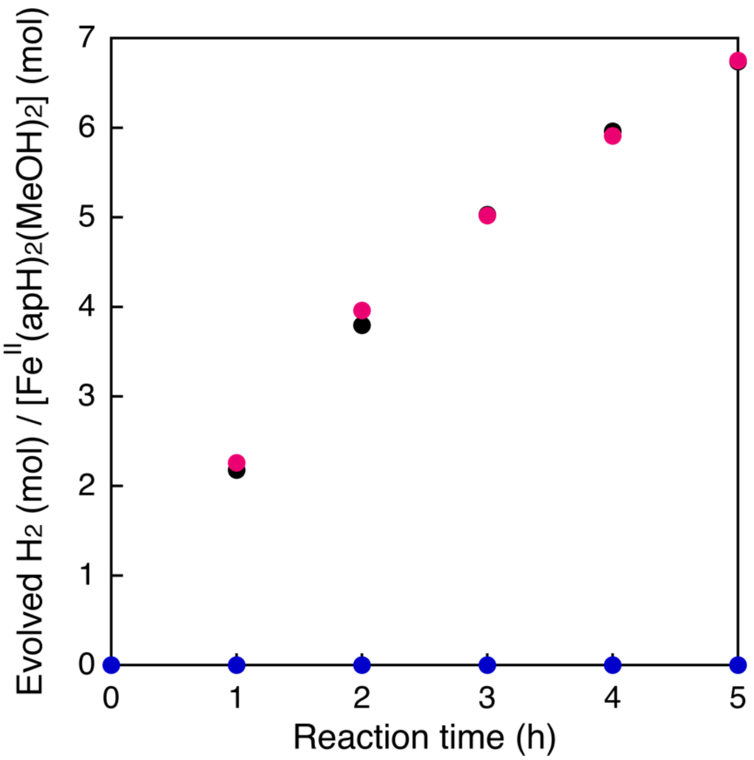

Supplementary Figure 10 | Temperature-dependent H_2 evolution from MeOH solutions of 1. Mols of evolved H_2 per mol of **1** as a function of reaction time for MeOH solutions of **1** (1 mM) under irradiation (289 ± 10 nm) at r.t. (black circles), under irradiation (289 ± 10 nm) at 45 °C (magenta circles); and under the exclusion of light at 45 °C (blue circles). Estimated relative standard deviation: 5%.

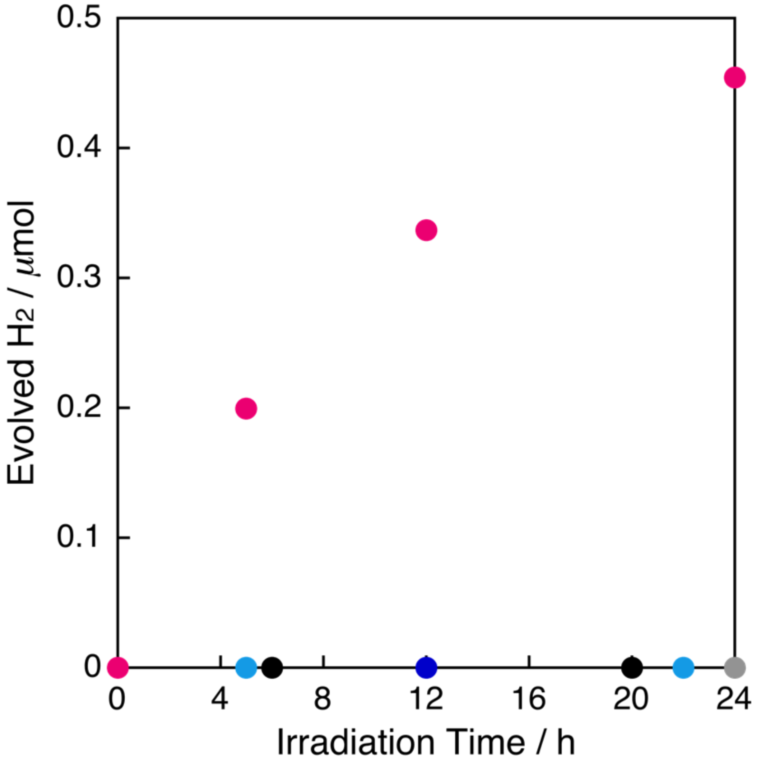

Supplementary Figure 11 | H_2 evolution from MeOH solutions of apH_2-based photocatalysts. Evolution of H_2 (μmol) as a function of PHER time (350 ± 10 nm; 21.8 mW) of MeOH solutions of apH_2 (2 mM; black circles), apH^- (2 mM; turquoise circles), **1** (1 mM; magenta circles), and $Fe^{II}(ClO_4)_2{\cdot}6H_2O$ (1 mM; blue circles), together with pure MeOH (gray circles). Estimated relative standard deviation: 5%.

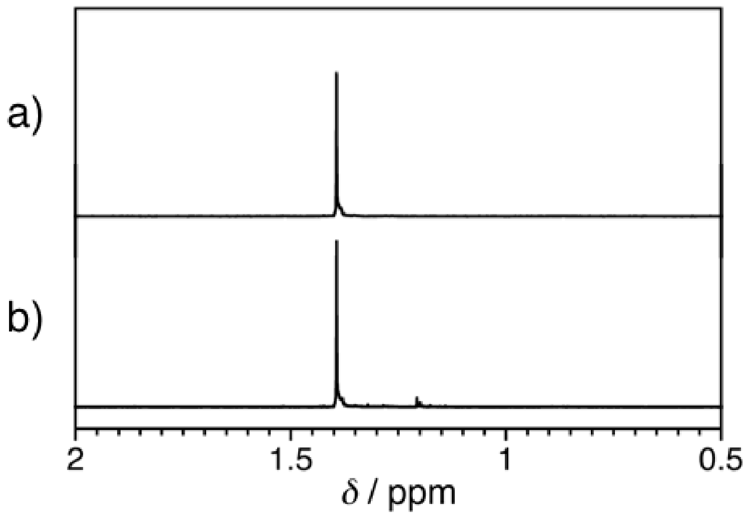

Supplementary Figure 12 | ^{1}H NMR spectra of *t*-BuSH. ^{1}H NMR spectrum of *t*-BuSH (2 mM) in MeOH-d_4 (**a**) prior and (**b**) posterior to photoirradiation at 289 ± 10 nm (24 h).

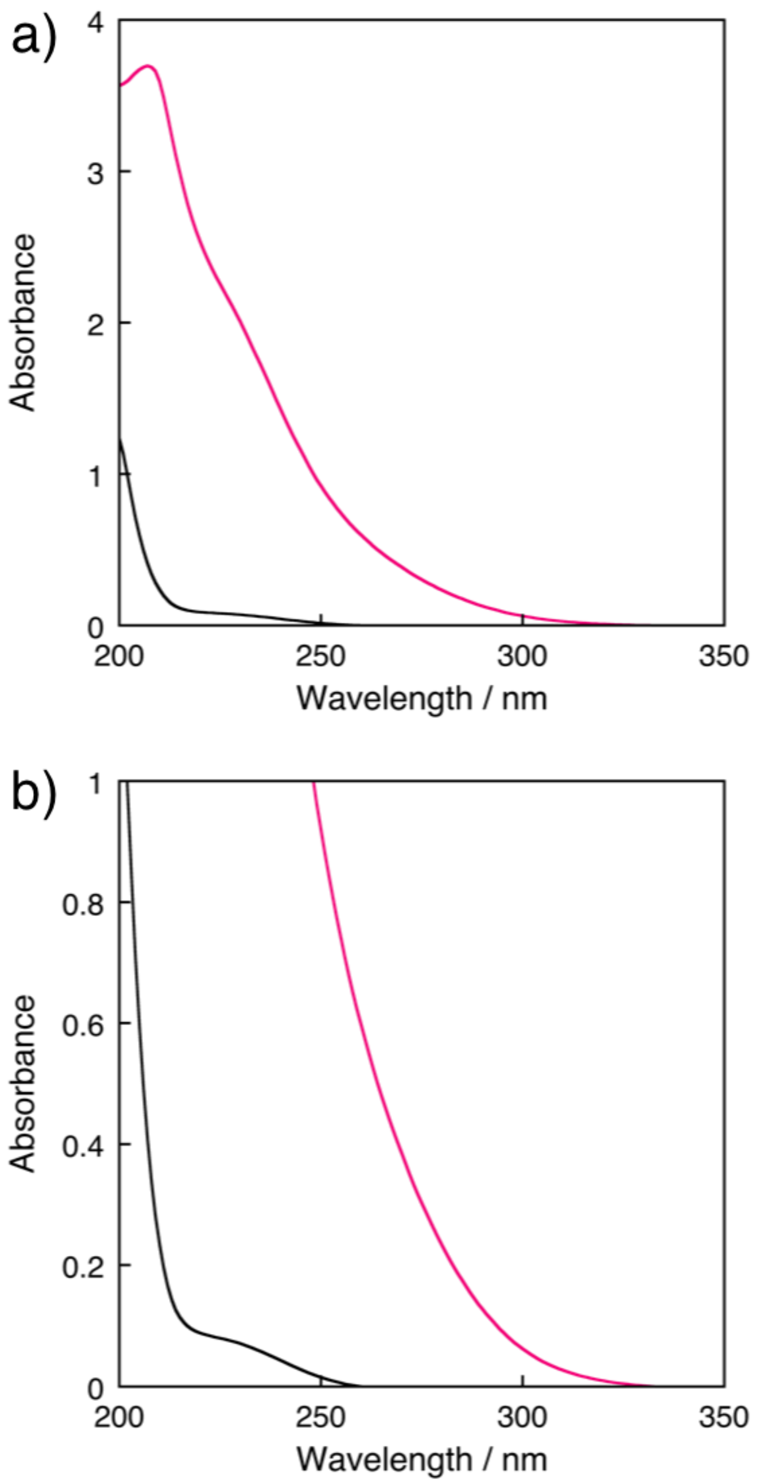

Supplementary Figure 13 | UV-vis-NIR spectra of *t*-BuSH and *t*-Bu_2S_2. (**a**) UV-vis spectra of *t*-BuSH (20 mM; black line) and *t*-Bu_2S_2 (20 mM; magenta line) in MeOH, and (**b**) magnification of the spectra (absorbance = 0-1).

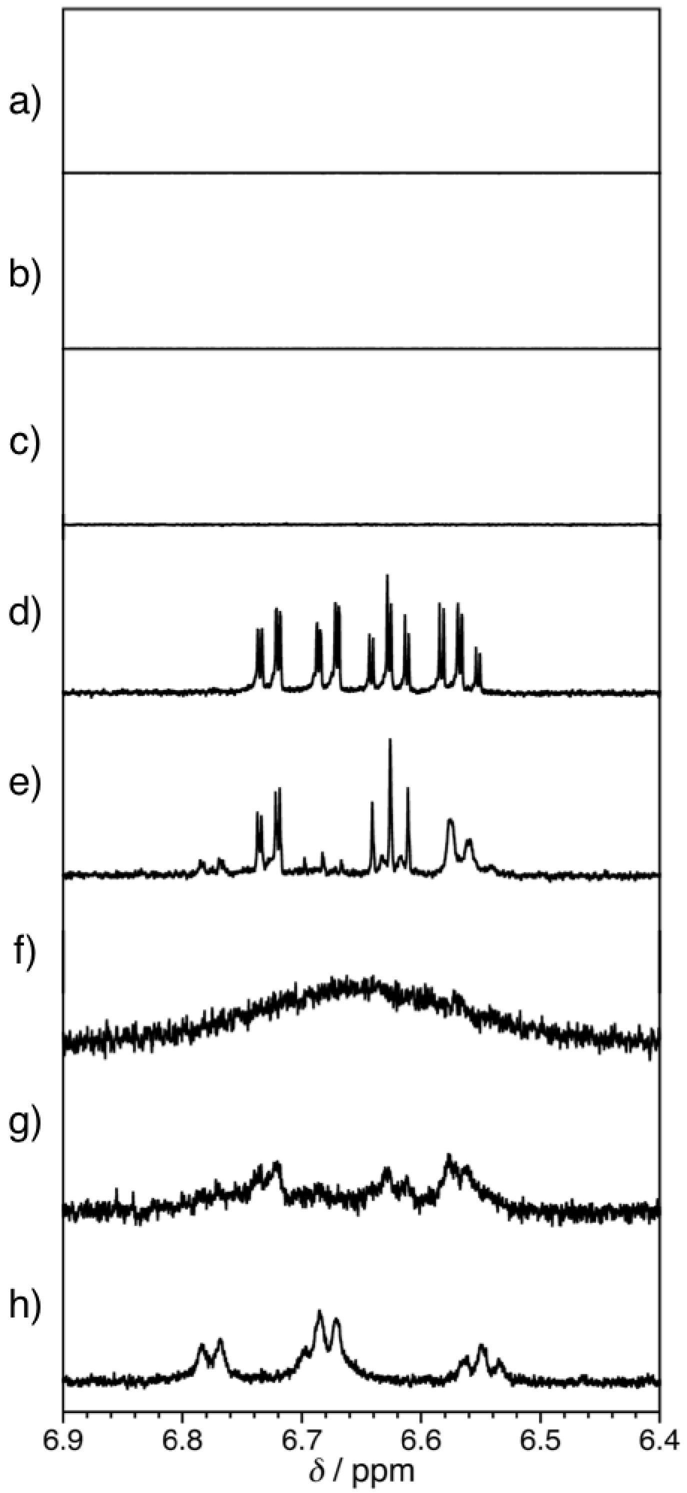

Supplementary Figure 14 | ^{1}H NMR spectra of apH$_2$-based photocatalysts with *t*-BuSH. ^{1}H NMR spectra in MeOH-d_4 in the aromatic region: (**a**) *t*-BuSH, (**b**) *t*-Bu$_2$S$_2$, (**c**) *t*-Bu$_2$S$_2$ after photoirradiation at 289 ± 10 nm (5 h), (**d**) *t*-BuSH with apH$_2$, (**e**) *t*-BuSH with apH$_2$ after photoirradiation at 289 ± 10 nm (5 h), (**f**) *t*-BuSH with **1**, (**g**) *t*-BuSH with **1** after photoirradiation at 289 ± 10 nm (5 h), and (**h**) *t*-BuSH with **1** after photoirradiation at 350 ± 10 nm (24 h).

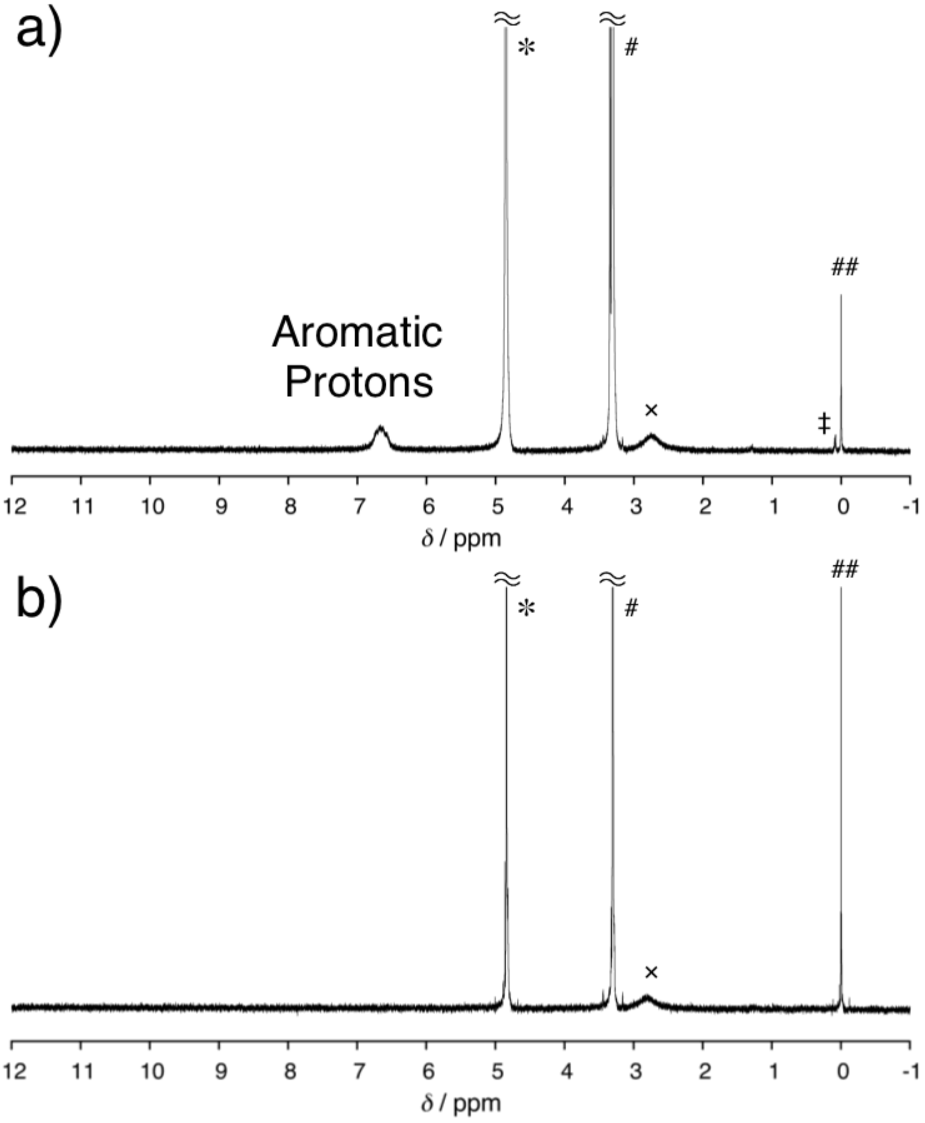

Supplementary Figure 15 | ^{1}H NMR spectra of 1 and MeOH-*d*$_4$. ^{1}H NMR spectrum of **1** in MeOH-*d*$_4$ at room temperature. ^{1}H NMR spectra of (**a**) **1** in MeOH-*d*$_4$ and of (**b**) pure MeOH-*d*$_4$. Signals marked with *, #, ×, ‡, and ## refer to H_2O, MeOH, a contaminating impurity in MeOH-*d*$_4$, silicon grease, and TMS, respectively.

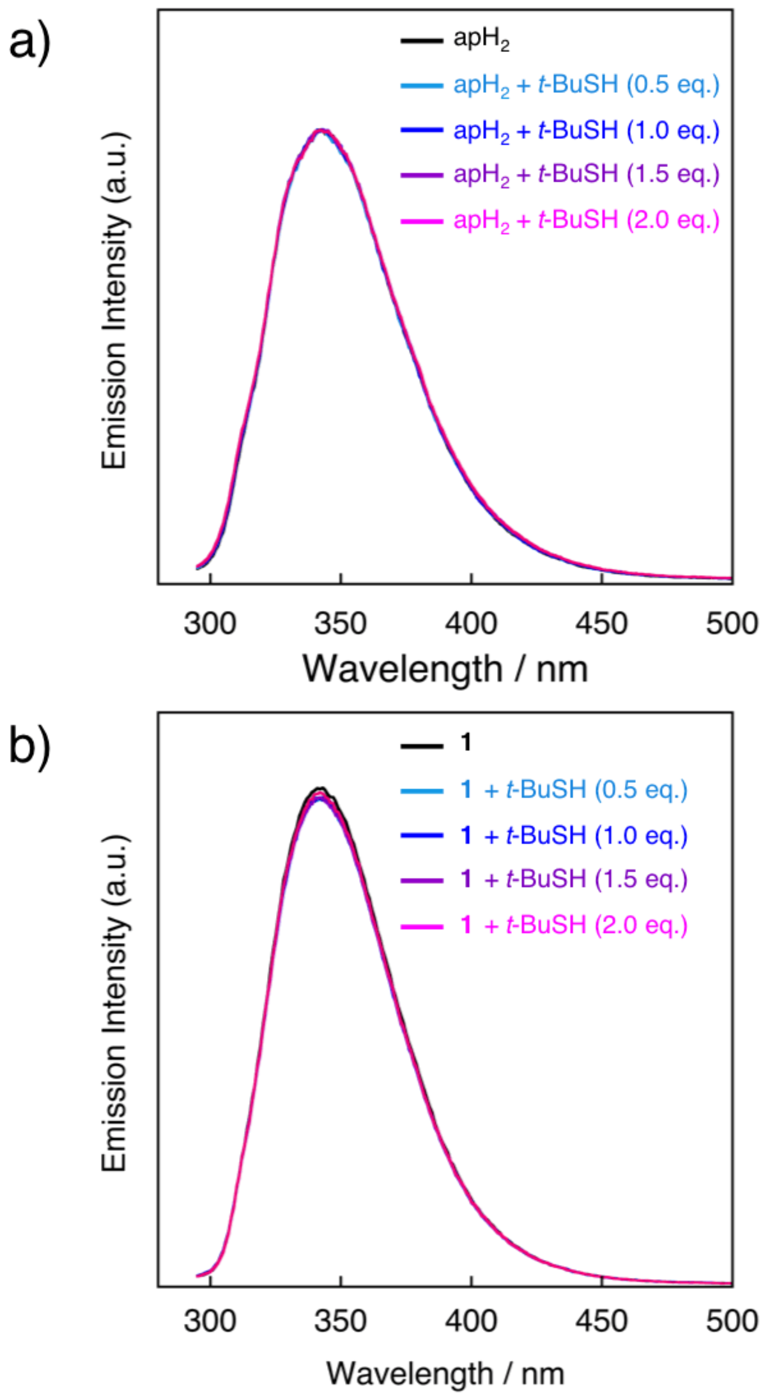

Supplementary Figure 16 | Emission spectra of apH$_2$ and 1. Emission spectra of (**a**) apH$_2$ (0.02 mM, black line) and (**b**) **1** (0.01 mM, black line) excited at 285 nm, together with those after addition of 0.5 (turquoise lines), 1.0 (blue lines), 1.5 (purple lines), and 2.0 (magenta lines) eq. of *t*-BuSH in MeOH under an atmosphere of N_2.

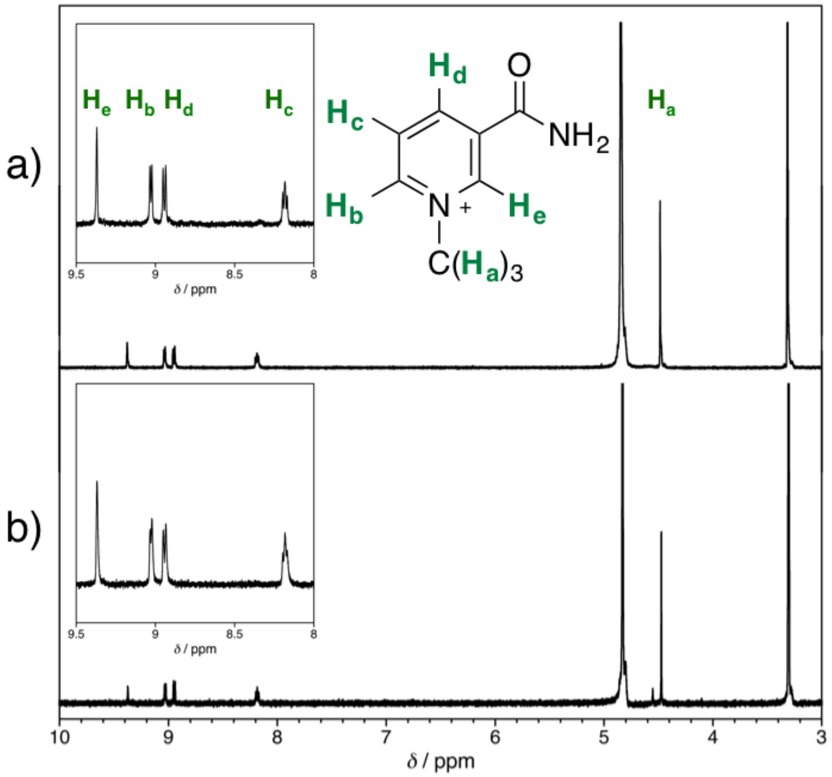

Supplementary Figure 17 | ^{1}H NMR spectra of 1 in the presence of NADCl. ^{1}H NMR spectra of **1** (1 mM) in the presence of NADCl (2 mM) in MeOH-d_4 (**a**) prior and (**b**) posterior to photoirradiation at 350 ± 10 nm (24 h). The insets show a magnification of the 9.5-8 ppm region. The amide protons could not be detected, most likely on account of an H/D exchange between NADCl and MeOH-d_4.

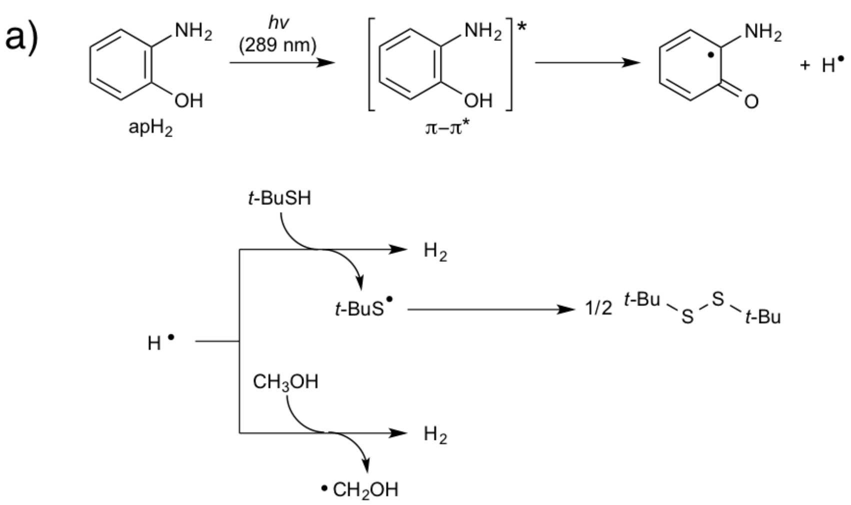

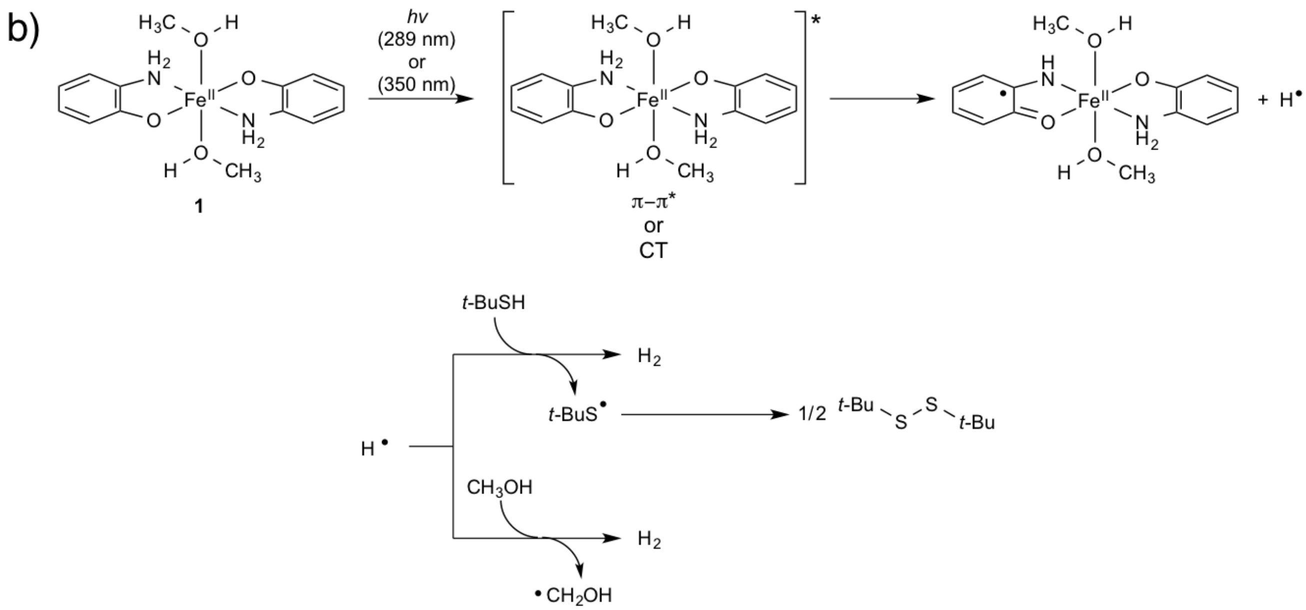

Supplementary Figure 18 | Plausible mechanism. Plausible mechanisms for the photochemical reactions of (**a**) apH_2 and (**b**) **1** in the presence of *t*-BuSH.

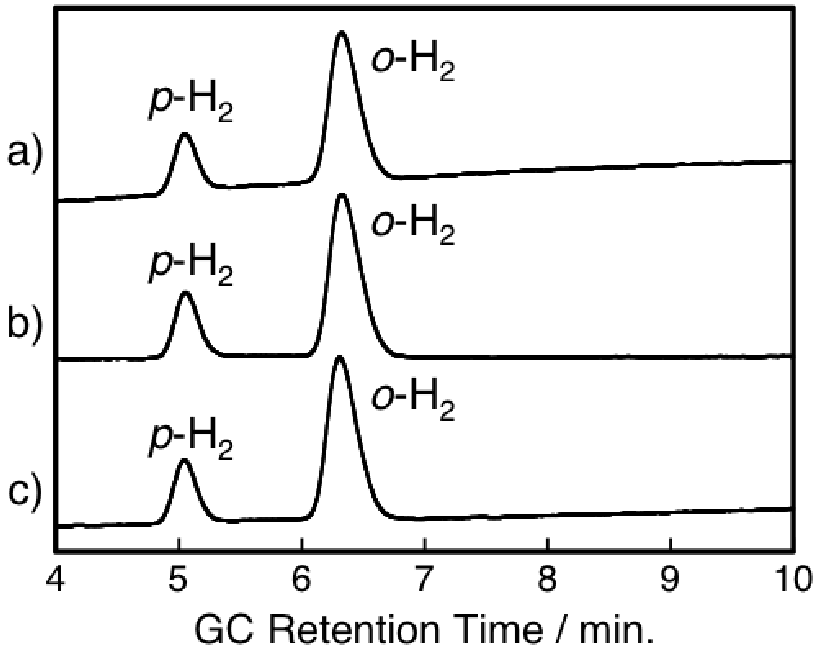

Supplementary Figure 19 | Gas chromatograms for standards. Gas chromatograms for standards: (**a**) H_2, (**b**) H_2 generated by treatment of CD_3OH (99.5%) with sodium metal, and (**c**) H_2 generated by the treatment of CD_3OH (99.5%) with $NaBH_4$.

Supplementary Table 1 | Quantum yields (Φ_{H2}) of previously reported catalysts for the photochemical dehydrogenation of MeOH

Catalyst	Φ_{H2} (%)[b]	Solvent	Photosensitizer	λ (nm)	T (°C)	Ref.
**1**	4.8[a]	MeOH	–	289±10	r.t.	This
	0.019[a]	MeOH	–	350±10	r.t.	This
apH^-	3.7[a]	MeOH	–	289±10	r.t.	This
apH_2	2.9[a]	MeOH	–	289±10	r.t.	This
cis-$Rh_2Cl_2(CO)_2(dpm)_2$ [b]	36.2[c]	MeOH	Acetone	286	20	2
	0.72	MeOH	–	260	20	2
$Pd_2Cl_2(dpm)_2$ [b]	–[d]	MeOH	Acetone	–[d]	64	3
$Et_4N[IrH(SnCl_3)_5]$	–[d]	MeOH	–	–[d]	65	4
$RuH_2(N_2)(PPh_3)_3$	–[d]	MeOH[e]	–	–[d]	150	5
$RuH_2(PPh_3)_3$	–[d]	MeOH[e]	–	–[d]	150	5
$Rh(H)(CO)(P^iPr_3)_2$	–[d]	MeOH	–	–[d]	–[d]	6

[a] estimated rsd: 5%. [b] dpm = bis(diphenylphosphino)methane. [c] Φ involves contributions of the photosensitizer to the photoreaction. [d] not mentioned. [e] [NaOH] = 1 M.

Supplementary Table 2 | Summary of the UV-vis-NIR spectroscopic data for apH$_2$-based photocatalysts and 2 in MeOH solution and in the solid state.

Compound	State	λ / nm (ε / M^{-1} cm^{-1})				
apH$_2$	Solution	230 (6,260)	285 (3,020)			
apH$^-$	Solution	230 (5,540)	288 (2,630)			
**1**	Solid^a	235^{sh}	287	335^{sh}	450^{sh}	750^{sh}
**1**	Solution	230 (17,690)	285 (8,550)	335 (1,210)^{sh}	450 (480)^{sh}	680 (118)^{sh}
**2**	Solid^a	235^{sh}	293	340		
**2**	Solution	227 (17,400)	283 (8,800)	335 (440)^{sh}	450 (130)^{sh}	607 (80)

^{a} KBr disk. ^{sh} shoulder peak.

Supplementary Table 3 | Crystallographic data for 1 and 2

Formula	$C_{14}H_{20}FeN_2O_4$	$C_{12}H_{14}CuN_2O_3$
FW	336.17	297.80
Crystal size (mm^3)	0.40 × 0.19 × 0.13	0.20 × 0.12 × 0.02
Crystal system	monoclinic	triclinic
Space group	$P2_1/c$ (No. 14)	P-1 (No. 2)
a (Å)	8.889(9)	9.666(4)
b (Å)	5.114(5)	9.702(4)
c (Å)	16.29(2)	12.945(5)
α (°)	90	82.884(13)
β (°)	100.40(2)	80.779(14)
γ (°)	90	89.707(18)
V (Å^3)	728.4(12)	1189.0(8)
T (K)	93	200
Z	2	4
D_{calc} (g cm^{-3})	1.533	1.664
F (000)	352.00	612.00
μ(Mo Kα) (cm^{-1})	10.513	18.381
Measured reflections	3873	6102
Unique reflections	1604	4900
Refined parameters	16.54	326
GOF on F^2	1.194	1.240
R_{int}	0.0468	0.0410
$R_1{}^a$	0.0576	0.0633
$wR_2{}^b$ (all data)	0.1559	0.1685

^{a} $R_1 = \Sigma||F_o| - |F_c||/\Sigma|F_o|$. ^{b} $wR_2 = \{[\Sigma w(F_o{}^2 - F_c{}^2)^2]/[\Sigma w(F_o{}^2)^2]\}^{1/2}$.

Supplementary Table 4 | Selected bond distances (Å) for 1 and 2, as well as for previously reported apH_2.

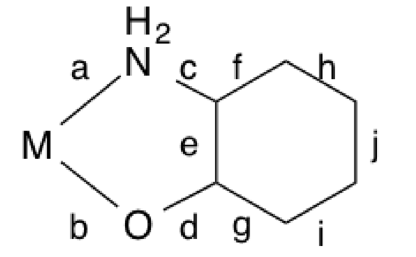

		Bond distances [Å]				
		a	b	c	d	e
**1**	L1	2.214(4)	2.048(3)	1.461(5)	1.342(5)	1.417(6)
**2**	L1	2.011(3)	1.940(3)	1.448(5)	1.329(4)	1.413(4)
	L2	2.013(3)	1.939(3)	1.454(5)	1.342(4)	1.408(6)
	L3	1.999(3)	1.939(3)	1.455(5)	1.335(4)	1.404(5)
	L4	2.015(3)	1.946(3)	1.450(5)	1.339(4)	1.402(5)
	Av.	2.010(3)	1.941(3)	1.452(5)	1.336(4)	1.407(5)
apH_2[a]				1.413(1)	1.366(2)	1.410(2)
		f	g	h	i	j
**1**	L1	1.385(7)	1.401(5)	1.390(6)	1.394(7)	1.408(6)
**2**	L1	1.376(5)	1.402(5)	1.384(5)	1.391(5)	1.393(5)
	L2	1.384(5)	1.397(5)	1.381(5)	1.381(5)	1.397(6)
	L3	1.379(5)	1.408(6)	1.399(6)	1.390(5)	1.371(7)
	L4	1.389(5)	1.404(6)	1.393(6)	1.389(5)	1.388(5)
	Av.	1.382(5)	1.403(6)	1.389(6)	1.388(5)	1.387(6)
apH_2[a]		1.397(2)	1.389(2)	1.398(2)	1.400(2)	1.397(2)

[a] Ref. 7.

Supplementary Table 5 | GC peak area for H_2 obtained from the treatment of CD_3OH with sodium metal, and from the treatment of CD_3OH with $NaBH_4$.

H_2 source	Area for *p*-H_2 × 10^3	Area for *o*-H_2 × 10^3	*o*-H_2 / *p*-H_2
Standard H_2	2.23	7.56	3.39
CD_3OH + Na	2.47	8.16	3.30
CD_3OH + $NaBH_4$	2.33	7.81	3.35

Supplementary Note 1.

Mixing two equivalents of a methanolic solution of apH_2 with an aqueous solution of $Cu^{II}(OAc)_2 \cdot H_2O$under an atmosphere of N_2 resulted in the formation of **2** as an aqua-blue solid. The unit cell of **2** contains two crystallographically independent mononuclear units, **A** and **B** (Supplementary Figures 5a and 5b). In these, the Cu atoms adopt a square-pyramidal coordination geometry, wherein the two bidentate NO ligands occupy the equatorial positions, while one O atom resides on the apical position. The structures of **A** and **B** are almost identical, except for their chirality around the Cu centre. The C–N (1.448-1.455(5) Å) and C–O (1.329-1.342(4) Å) bond distances in the four bidentate NO ligands indicate a single bond character for these bonds, and the C–C bond distances in the six-membered rings (1.371-1.413(7) Å) suggest high levels of aromaticity. As in the case of **1**, these results suggest that the ligands in **2** adopt a structure that is consistent with apH^-.[8,9] As the Cu–O (**A**: Cu1–O3 = 2.431(3) Å, **B**: Cu2–O6 = 2.424(3) Å) bond distances are consistent with those of previously reported Cu^{II}–OH_2 bonds (2.475(10) Å) in *e.g.* *cis*-[Cu^{II}(L-isoleucinato)$_2$(H_2O)],[10] the apical ligand in **2** should be assigned as H_2O, leading to the formulation of **2** as *trans*-[$Cu^{II}(apH_2)_2(H_2O)$]. The difference in coordination number between **1** and **2** should be attributed to the different degree of electron repulsion around the metal centre between Fe^{II} (d^6) and Cu^{II} (d^9). As in the case of **1**, the observed dihedral angles (0.19, 0.93, 10.42, and 11.76°) between the O–Cu–N and O–C–C–N planes in apH^- in **2** indicate interactions between the d-orbitals on Cu^{II} and the π-orbitals on apH^-. Although antibacterial activity has been demonstrated for apH^- complexes of

Cu(II), their detailed structures have not yet been reported.[11-13] Complex **2** thus represents the first example of a structurally resolved apH^- complex of Cu(II).

The UV-vis-NIR spectrum of **2** in solution (1.00 mM in MeOH) exhibited five absorption bands at λ_{max} (ε / M^{-1} cm^{-1}) = 227 (17,400), 283 (8,800), 335 (440), 450 (130), and 607 nm (80) (Supplementary Table 2 and Figure 6). The two absorption bands at 227 and 283 nm with large molar extinction coefficients are similar to those of **1**, apH_2, and apH^-, and were accordingly assigned to transitions involving apH^--centred π–π* transitions. Conversely, the three bands at 335, 460, and 607 nm, which were not observed for apH^- and apH_2, should be assigned to CT transitions between Cu(II) and the apH^- ligands (335 and 460 nm), as well as to Cu-centred d-d transitions (607 nm).

No time-dependent spectral change was observed for **1** (Supplementary Figure 7a), whereas the spectral profile of **2** gradually changed over the course of 20 h under an atmosphere of N_2 (Supplementary Figure 7b), resulting in the emergence of an absorption band at 427 nm. The absorption maximum of this band was identical to that of 2-aminophenoxazine-3-one (APX), thus indicating the formation of APX from **2** in MeOH. This notion is supported by previous reports on APX analogs such as actinomycin D, which can be synthesized from the appropriate *o*-aminophenol precursors by phenoxazine synthase-catalyzed oxidative deprotonation.[14] Furthermore, Nishinaga and co-workers reported the Co^{II}(salen)-catalyzed (salen = *N*,*N'*-bis(salicylidene)ethylenediamine) formation of APX from apH_2, and proposed a nucleophilic attack from the amino group of apH_2 to the Co^{II}-bound benzoquinoimine intermediate as the initial step of the reaction, followed by a multistep

oxidative deprotonation and the formation of a C–O bond.[1] Another previous report rationalized the formation of APX from **2** on the basis of the generation of a Cu-bound benzoquinoimine intermediate (Supplementary Figure 8). As the reduction potential of Cu^{II} is in general more positive than that of Fe^{II}, the different reactivity of **1** relative to **2** might also be related to the increased electron-accepting capacity of Cu^{II} in **2** compared to that of Fe^{II} in **1**. These results suggest a remarkable influence of the metal centre on the electron-donating ability of the apH^{-} ligand, and the nature of the metal ion should thus have a fundamental impact on the PHER activity of apH^{-} systems. As **2** is not stable in MeOH, no further investigations were carried out.

Supplementary Methods.

Crystallographic Data Collection and Structure Refinement. Single-crystal X-ray diffraction patterns were recorded on a Rigaku VariMax with Saturn equipment (**1**), or on a Rigaku AFC-7R diffractometer, equipped with a Mercury CCD area detector (**2**). In both cases, graphite-monochromated Mo-Kα radiation (λ = 0.71069 Å) was used. Single crystals of suitable size and quality were selected under paraffin oil or silicon grease, and mounted onto MicroMounts (MiTeGen), and cooled to 93 (**1**) or 200 K (**2**) using an N_2 flow-type temperature controller. Molecular structures were solved by direct methods (SIR2004),[15] which allowed the successful location of all non-hydrogen atoms within the unit cell. All calculations were carried out using the CrystalStructure software package,[16] except for refinement calculations, which were carried out using SHELXL-97.[17] A summary of the crystallographic data for **1** and **2** is shown in Supplementary Table 3. Full crystallographic details were deposited at the Cambridge Crystallographic Data Centre (CCDC) under supplementary publication numbers CCDC-1062112 (**1**) and CCDC-1418535 (**2**).

Supplementary References.

1 Maruyama, K., Moriguchi, T., Mashino, T. & Nishinaga, A. Highly selective formation of 2-aminophenoxazin-3-one by catalytic oxygenation of *o*-aminophenol. *Chem. Lett.* **25,** 819-820, (1996).

2 Takahashi, T., Shinoda, S. & Saito, Y. The mechanisms of photocatalytic dehydrogenation of methanol in the liquid phase with *cis*-$[Rh_2Cl_2(CO)_2(dpm)_2]$ complex catalyst. *J. Mol. Catal.* **31**, 301-309, (1985).

3 Yamamoto, H., Shinoda, S. & Saito, Y. Photocatalytic dehydrogenation of methanol in the liquid phase with *cis*-$Rh_2Cl_2(CO)_2(dpm)_2$ and $Pd_2Cl_2(dpm)_2$ complex catalysts. *J. Mol. Catal.* **30,** 259-266, (1985).

4 Makita, K., Nomura, K. & Saito, Y. Photocatalytic dehydrogenation of methanol using $[IrH(SnCl_3)_5]^{3-}$ complex. *J. Mol. Catal.* **89,** 143-149, (1994).

5 Morton, D. & Cole-Hamilton, D. J. Molecular hydrogen complexes in catalysis: highly efficient hydrogen production from alcoholic substrates catalysed by ruthenium complexes. *J. Chem. Soc., Chem. Commun.* 1154-1156, (1988).

6 Delgado-Lieta, E., Luke, M. A., Jones, R. F. & Cole-Hamilton, D. J. The photochemical decomposition of alcohols catalyzed by tri(isopropyl) phosphine complexes of rhodium(I). *Polyhedron* **1**, 839-840, (1982).

7 Allen, F. H. *et al.* Crystal engineering and correspondence between molecular and crystal structures. Are 2- and 3-aminophenols anomalous? *J. Am. Chem. Soc.* **119**, 3477-3480, (1997).

8 Matsumoto, T. *et al.* Nonprecious-metal-assisted photochemical hydrogen production from *ortho*-phenylenediamine. *J. Am. Chem. Soc.* **135,** 8646-8654, (2013).

9 Reynolds, M. *et al.* 4-Nitrocatechol as a probe of a Mn(II)-dependent extradiol-cleaving catechol dioxygenase (MndD): comparison with relevant Fe(II) and Mn(II) model complexes. *J. Biol. Inorg. Chem.* **8,** 263-272, (2003).

10 Weeks, C. M., Cooper, A. & Norton, D. A. The crystal structure of the copper(II) complex of L-isoleucine. *Acta Crystallogr. Sect. B* **25,** 443-450, (1969).

11 Loginova, N. V. *et al.* Synthesis and biological evaluation of copper (II) complexes of sterically hindered *o*-aminophenol derivatives as antimicrobial agents. *Bioorg. Med. Chem. Lett.* **16,** 5403-5407, (2006).

12 Loginova, N. V. *et al.* Redox-active antifungal cobalt(II) and copper(II) complexes with sterically hindered *o*-aminophenol derivatives. *Polyhedron* **27,** 985-991, (2008).

13 Loginova, N. V. *et al.* Redox-active metal(II) complexes of sterically hindered phenolic ligands: antibacterial activity and reduction of cytochrome c. Part II. Metal(II) complexes of *o*-diphenol derivatives of thioglycolic acid. *Polyhedron* **30,** 2581-2591, (2011).

14 Hollstein, U. Actinomycin. Chemistry and mechanism of action. *Chem. Rev.* **74,** 625-652, (1974).

15 Burla, M. C. *et al.* SIR2004: an improved tool for crystal structure determination and refinement. *J. Appl. Crystallogr.* **38,** 381-388, (2005).

16 CrystalStructure 3.8.2, Crystal Structure Analysis Package; Rigaku and Rigaku/MSC: The Woodlands, TX, 2000−2006.

17 Sheldrick, G. SHELX-97 Program for crystal structure solution and the refinement of crystal structures, Institut für Anorganische Chemie der Universität Göttingen, Tammanstrasse 4, D-3400 Göttingen, Germany, 1997.
